# Targeted transcriptomics reveals signatures of large-scale independent origins and concerted regulation of effector genes in *Radopholus similis*

**DOI:** 10.1371/journal.ppat.1010036

**Published:** 2021-11-08

**Authors:** Paulo Vieira, Roxana Y. Myers, Clement Pellegrin, Catherine Wram, Cedar Hesse, Thomas R. Maier, Jonathan Shao, Georgios D. Koutsovoulos, Inga Zasada, Tracie Matsumoto, Etienne G. J. Danchin, Thomas J. Baum, Sebastian Eves-van den Akker, Lev G. Nemchinov

**Affiliations:** 1 USDA-ARS Molecular Plant Pathology Laboratory, Beltsville, Maryland, United States of America; 2 School of Plant and Environmental Sciences, Virginia Tech, Blacksburg, Virginia, United States of America; 3 Daniel K. Inouye U.S. Pacific Basin Agricultural Research Center, USDA ARS, Hilo, Hawaii, United States of America; 4 Department of Plant Sciences, University of Cambridge, Cambridge, United Kingdom; 5 USDA-ARS Horticultural Crops Research Unit, Corvallis, Oregon, United States of America; 6 Department of Plant Pathology and Microbiology, Iowa State University, Ames, Iowa, United States of America; 7 INRAE, Université Côte d’Azur, CNRS, Institute Sophia Agrobiotech, Sophia Antipolis, France; UNITED STATES

## Abstract

The burrowing nematode, *Radopholus similis*, is an economically important plant-parasitic nematode that inflicts damage and yield loss to a wide range of crops. This migratory endoparasite is widely distributed in warmer regions and causes extensive destruction to the root systems of important food crops (e.g., citrus, banana). Despite the economic importance of this nematode, little is known about the repertoire of effectors owned by this species. Here we combined spatially and temporally resolved next-generation sequencing datasets of *R*. *similis* to select a list of candidates for the identification of effector genes for this species. We confirmed spatial expression of transcripts of 30 new candidate effectors within the esophageal glands of *R*. *similis* by *in situ* hybridization, revealing a large number of pioneer genes specific to this nematode. We identify a gland promoter motif specifically associated with the subventral glands (named Rs-SUG box), a putative hallmark of spatial and concerted regulation of these effectors. Nematode transcriptome analyses confirmed the expression of these effectors during the interaction with the host, with a large number of pioneer genes being especially abundant. Our data revealed that *R*. *similis* holds a diverse and emergent repertoire of effectors, which has been shaped by various evolutionary events, including neofunctionalization, horizontal gene transfer, and possibly by *de novo* gene birth. In addition, we also report the first GH62 gene so far discovered for any metazoan and putatively acquired by lateral gene transfer from a bacterial donor. Considering the economic damage caused by *R*. *similis*, this information provides valuable data to elucidate the mode of parasitism of this nematode.

## Author summary

*Radopholus similis* is considered to be among the top 10 most damaging plant-parasitic nematodes world-wide. The molecular dialogue established by plant-parasitic nematodes and the host plant is driven by secreted nematode proteins, also known as effectors. Here we use targeted transcriptomics to predict and validate the most comprehensive set of effectors for this plant-parasitic nematode. Contrary to long-held assumptions that migratory nematodes are less specialized plant-parasitic nematodes, we showed that the repertoire of effectors of this migratory nematode is largely complex and novel. Among the 30 new effector genes identified, 19 were pioneer genes without *a priori* putative function or annotation but highly expressed during plant interaction. The identification of a promoter motif element for a significant set of these effectors suggest a concerted regulation of these genes by the nematode. The increase knowledge of the effector repertoire of this economic important species not only contributes to the understanding of the mechanisms of parasitism employed by migratory nematodes, but will also allow the development of effector gene targeted strategies to control this nematode.

## Introduction

The burrowing nematode *Radopholus similis* is an important plant-pathogen that inflicts damage and yield losses to a broad range of crops. This species is considered to be among the top 10 most damaging plant-parasitic nematodes world-wide [1]. Although this migratory endoparasite is widely known for its substantial economic impact on food crops, such as banana, citrus and pepper, this species has a host range that extends to more than 300 plant species, including coconut, coffee, sugarcane, tea, as well as ornamental plants [2]. *Radopholus similis* is widely distributed in warm subtropical and tropical regions, including South America, the Caribbean, Africa, Asia, and the Pacific Islands [2,3]. In addition, this nematode has been recurrently found in temperate regions (e.g., Europe) as a consequence of transporting infested plant material, causing noteworthy problems on ornamental glasshouse crops [4]. In Hawaii, for example, *R*. *similis* is the most common plant-parasitic nematode recovered on the island, causing substantial damage to the production of banana and *Anthurium* [5]. Infected anthuriums can become severely stunted resulting in smaller flowers and yield reductions of up to 50%. *Radopholus similis* is of great economic importance in commercial banana plantations as the causative agent of “toppling” disease impacting an important food resource of the people living in these areas [5,6].

*Radopholus similis* is an obligate parasite that feeds from the roots of living plants. All life stages of the nematode can be found inside the roots. The life cycle of *R*. *similis* is punctuated by six developmental stages: eggs, four juvenile stages (J1-J4), and adults (males and females). Although sexual reproduction can occur in this species, in the absence of males, females become hermaphrodites, assuring the continuous reproduction of the nematode [7]. Similar to other migratory endoparasitic nematodes, all stages are vermiform and capable of migrating within the roots (except eggs and J1). All migratory stages are able to feed from the roots, with the exception of males, which possess a degenerated stylet and do not feed [8]. *Radopholus similis* feeds mainly from the cortex root cells, resulting in extensive lesions and cavities along the root system of the plant [9,10]. The severe damage to the roots reduces water and nutrient uptake by the plant, and consequently impairs growth and plant development [2,5].

The inclusion of *R*. *similis*, together with other root lesion nematodes (i.e., *Pratylenchus* spp.), in the family Pratylenchidae is typically defined by their morphological features, which could be an outcome of convergent evolution related to their similar migratory parasitism strategies [4]. Like other root lesion nematodes, *R*. *similis* does not induce specialized feeding sites, such as giant-cells or syncytia normally induced by the sedentary root-knot (*Meloidogyne* spp.) or cyst (*Heterodera* and *Globodera* spp.) nematodes, respectively [11,12]. However, molecular analyses using different *loci* showed that the Pratylenchidae family is polyphyletic and that *R*. *similis* is a sister taxon and more closely related to cyst nematodes (Heteroderidae) rather than to other migratory Pratylenchidae [12–16].

Plant-parasitic nematodes comprise repertoires of secreted proteins (namely effectors) that interact with the host plant with a myriad of functions [17,18]. Nematode effector proteins are mainly produced by three specialized esophageal glands (one dorsal and two sub-ventral glands) and ultimately secreted through the nematode stylet into the plant tissues [17]. Depending on their mode-of-action, nematode effectors can be secreted into the apoplasm or directly into the host cell cytoplasm [19,20]. These secreted effectors facilitate host infection by manipulating host processes, suppressing host defenses and allowing nematodes to use the cell host contents as a source of nutrients [17,21].

The recent release of genome and transcriptome datasets for *R*. *similis* provide a general overview of the effector repertoire and evolutionary insights of this migratory nematode [12,22–24]. The majority of the candidate effectors reported so far for *R*. *similis* are part of the common panel of effectors often found for other migratory and sedentary nematodes [12,23,25]. This list includes genes belonging to different families of cell wall-degrading enzymes (CWDEs), which are often acquired by horizontal gene transfer (HGT) and potentially involved in degradation of host cell components assisting in penetration and migration of the nematode in root tissues [26–29]. Apart from genes related to cell wall modification, a set of other candidate effectors has been identified that are putatively involved in defense suppression, response to host oxidative stress, and other unknown functions [22,23]. Nevertheless, only a small number of *R*. *similis* effectors have been experimentally validated and functionally characterized in comparison to other agricultural important nematode species, such as cyst and root-knot nematodes [26,27,30–33].

The presence of a common set of effectors (excluding those potentially originated by HGT) indicates that some of these genes are evolutionarily conserved among different plant-parasitic nematodes [34]. Considering that a significant number of effector proteins are frequently evolutionarily diverse and generally lack recognizable homology in other species, the identification of effector proteins based only on sequence similarity could overlook the presence of key components of the effector repertoire for any single nematode species. This is consistent with the fact that most of the known effectors used as primary criterion of comparison are related to sedentary nematodes (e.g., cyst and root-knot nematodes), which also display poor overlap of the effector repertoires among each other [35]. This type of approach, although useful, may generate some bias when looking into the effector repertoires of distant and unrelated species, such as the case of strictly migratory plant-parasitic nematodes. Although migratory nematodes have been perceived as less specialized due to the absence of the induction of a feeding site [36], larger portfolios of effectors have been recently discovered for unrelated migratory species, such as the root lesion nematode *Pratylenchus penetrans* (Pratylenchidae [37,38]) and the pinewood nematode *Bursaphelenchus xylophilus* (Parasitaphelenchidae [39,40]). An important feature of these effector catalogues is the existence of a distinct number of secreted proteins without prior annotation or predictive function (i.e., pioneer genes) that are mostly genus- or species-specific [38,40]. Under this assumption, and despite the recent advances in the identification of effector genes in *R*. *similis* [12,22,23,26,27,30–33], the particular phylogenetic position of this species warrants further studies of the biology and nature of the effector repertoire of *R*. *similis*. Understanding the evolution of nematode effectors is imperative as this understanding comprises the proteins that drive the molecular dialogue with the host [25].

On the basis of genomic and targeted transcriptomic datasets, we provide here a comprehensive catalogue of effectors for *R*. *similis*. We confirmed spatial expression of 30 new effectors within the esophageal glands of *R*. *similis* by *in situ* hybridization. Furthermore, we identified new exclusive examples of HGT events to this species, including the first gene belonging to the glycosyl hydrolase 62 (GH62) family ever described for a metazoan. We then combined *in situ* localization of effectors and available genomic data to identify a non-coding motif associated with gene expression specific to the nematode esophageal glands (i.e., the Rs-SUB box). The motif is a hallmark of spatial and concerted regulation of these effectors and is specific to gene expression in the nematode subventral esophageal glands. Considering the economic damage caused by *R*. *similis*, this study offers critical information for the potential use of nematode-specific effector genes to be exploited as new targets for nematode control. It also provides valuable data to trace the evolutionary scenarios of the arsenal of effectors acquired by this particular species.

## Results and discussion

### *Radopholus similis* secretome prediction and identification of known candidate effector genes

Some nematode secreted proteins play key roles for successful invasion and establishment of nematodes within the host plant [17]. Using the genome sequence of *R*. *similis* (Rv population [24]) we identified putatively secreted proteins for this nematode. Out of the 14,817 unique transcripts, a total of 2,218 (14.9%) were predicted to encode putatively secreted proteins without transmembrane domains (TMHMM) (S1 Table). We then used PFAM domain searches and BLAST-based similarity searches against the non-redundant (nr) database at NCBI to annotate the full set of 2,218 putatively secreted proteins. A total of 1,221 (55%) were annotated by one of these databases, while 997 (45%) had no putative annotation (S1 Table).

**Fig 1:**
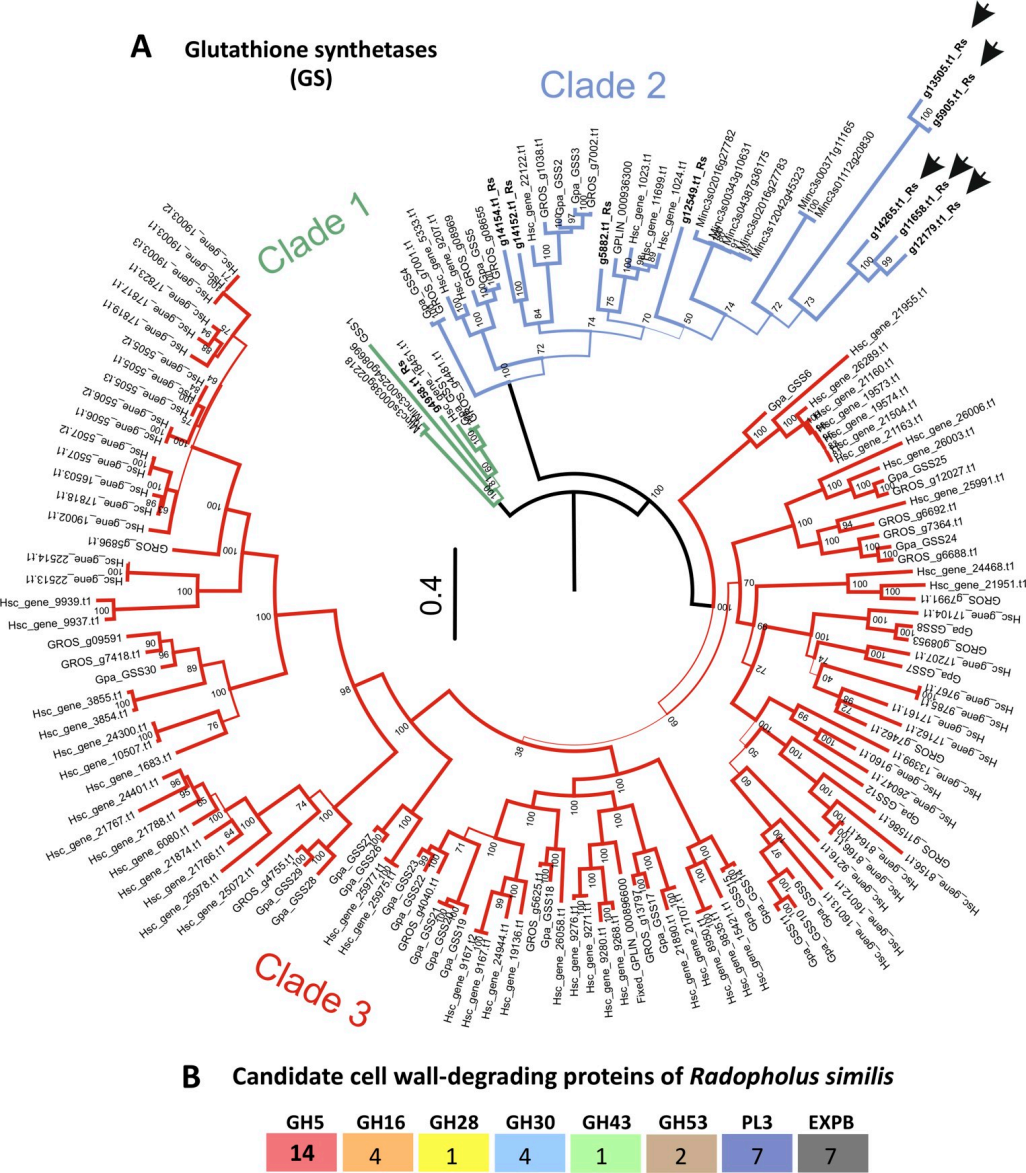
Identification of selected candidate effector gene families of *Radopholus similis*. **(A)** Maximum-likelihood phylogenetic tree of glutathione synthetase (GS) proteins of *R*. *similis* with GS sequences of other plant-parasitic nematodes (adapted from [41]). Clade 1 (green) contains the “progenitor” GS sequence of each nematode species included in the tree. Clade 2 (blue) represents the first expansion of GS genes in plant-parasitic nematodes and contains 9 GS coding genes from *R*. *similis*. Arrows indicate putative secreted GS-like effectors of *R*. *similis*. Clade 3 (red) represents the second GS genes expansion, and contains all known GS-like effectors of cyst and reniform nematodes. **(B)** Number of genes encoding putative cell wall-degrading proteins in *R*. *similis*. GH: glycosyl hydrolyse; PL: pectate lyase; EXPB: expansin-like.

To identify the *R*. *similis* homologues of previously described effectors in plant-parasitic nematodes, a targeted effector search was performed based on sequence similarity [either using BLAST or the presence of characteristic protein domains of previously described carbohydrate-active enzymes (CAZymes)]. Effectors common to many plant-parasitic nematodes with different strategies of parasitism were identified (S1 Table). They comprise, for example, chorismate mutases, fatty acid- and retinol-binding proteins, venom allergen-like proteins, SXP/RAL-2 proteins, several putative esophageal gland genes reported for both cyst and root-knot nematodes, and a wide range of secreted proteases and proteases inhibitors (S1 Table). While it is widely cited that effectors specific to sedentary nematodes, and potentially involved in the induction of a feeding site, are not detected within the genome of *R*. *similis* (highlighting the differences of parasitism strategies among migratory and sedentary nematodes), we noted a substantial number of glutathione synthetase (GS)-like effectors (n = 5) in *R*. *similis* (i.e., putatively secreted proteins with glutathione synthetase-like domains PF03917 and PF03199). The occurrence of GS-like effector genes outside the cyst and reniform nematodes is highly unusual. The GS gene family in plant-parasitic nematodes was generated by two large-scale gene expansion events separated by millions of years of evolution (Fig 1A), giving rise to three separate GS clades [41]. It has been reported that Clade 1 is conserved in nematodes and contains the progenitor house-keeping GS, Clade 2 only contains sequences from endoparasites (sedentary and migratory) but does not contain secreted proteins (or putative effectors), and Clade 3 is restricted to the cyst and reniform nematodes and contains all known GS-like effectors and the only secreted GS-like proteins ever described [41]. Five of the ten *R*. *similis* GS-like genes encode a signal peptide and are all grouped in a monophyletic subclade within Clade 2 (Fig 1A). The remaining *R*. *similis* GS genes are distributed across Clade 2 (n = 4) and Clade 1 (n = 1).

With regard to effectors that carry out cell wall-modifying activities in other species, a prominent set of putatively secreted CAZymes were identified in the secretome of the *R*. *similis* Rv population (S1 Table), consistent with previous data generated for other *R*. *similis* populations [12,23]. A total of 40 genes were distributed among the main CWDEs previously reported as components of the effectorome of other plant-parasitic nematodes (Fig 1B). These included ß-1,4-endoglucanases [glycosyl hydrolases (GH) 5], glycosyl hydrolases of family 16 (GH16), as well as candidates glucuronoarabinoxylan endo-1,4-ß-xylanases (GH30), arabinan endo-1,5-ß-L-arabinosidases (GH43), arabinogalactan endo-1,4-ß-galactosidase (GH53), pectate lyases (PL3), and expansin-like proteins often identified as multigenic families in diverse plant-parasitic nematodes (Fig 1B).

### Prediction of candidate effectors using a nematode esophageal gland library

Direct sequencing of mRNA extracted from the nematode esophageal glands can expedite the identification of effectors from both sedentary and migratory nematodes [38,40,42]. Therefore, to resolve a more comprehensive list of candidate effectors for *R*. *similis*, not restricted by homology to previous effector characterizations in related species, we took advantage of a ‘454’ mRNA library originated from the esophageal gland cells of this species [42]. All reads were mapped to the 14,817 predicted transcripts of the *R*. *similis* Rv genome. Using this approach, we detected the presence of 4,873 transcripts in the gland cell library. Next, and to increase the likelihood of identifying new candidate effectors, we focused our analyses on transcripts that also encode putatively secreted proteins without TMHMM within this library. This analysis identified a final set of 540 transcripts (S2 Table), representing 24.3% (540/2,218) of the total number of predicted secreted proteins (without TMHMM) in the entire *R*. *similis* Rv genome. A normalized read count (FPKM = fragments per kilobase per million mapped reads) was then used to rank all transcripts in agreement to their relative abundance in the gland cell library, ranging from 1.92 < FPKM < 57,141 (S2 Table).

**Fig 2:**
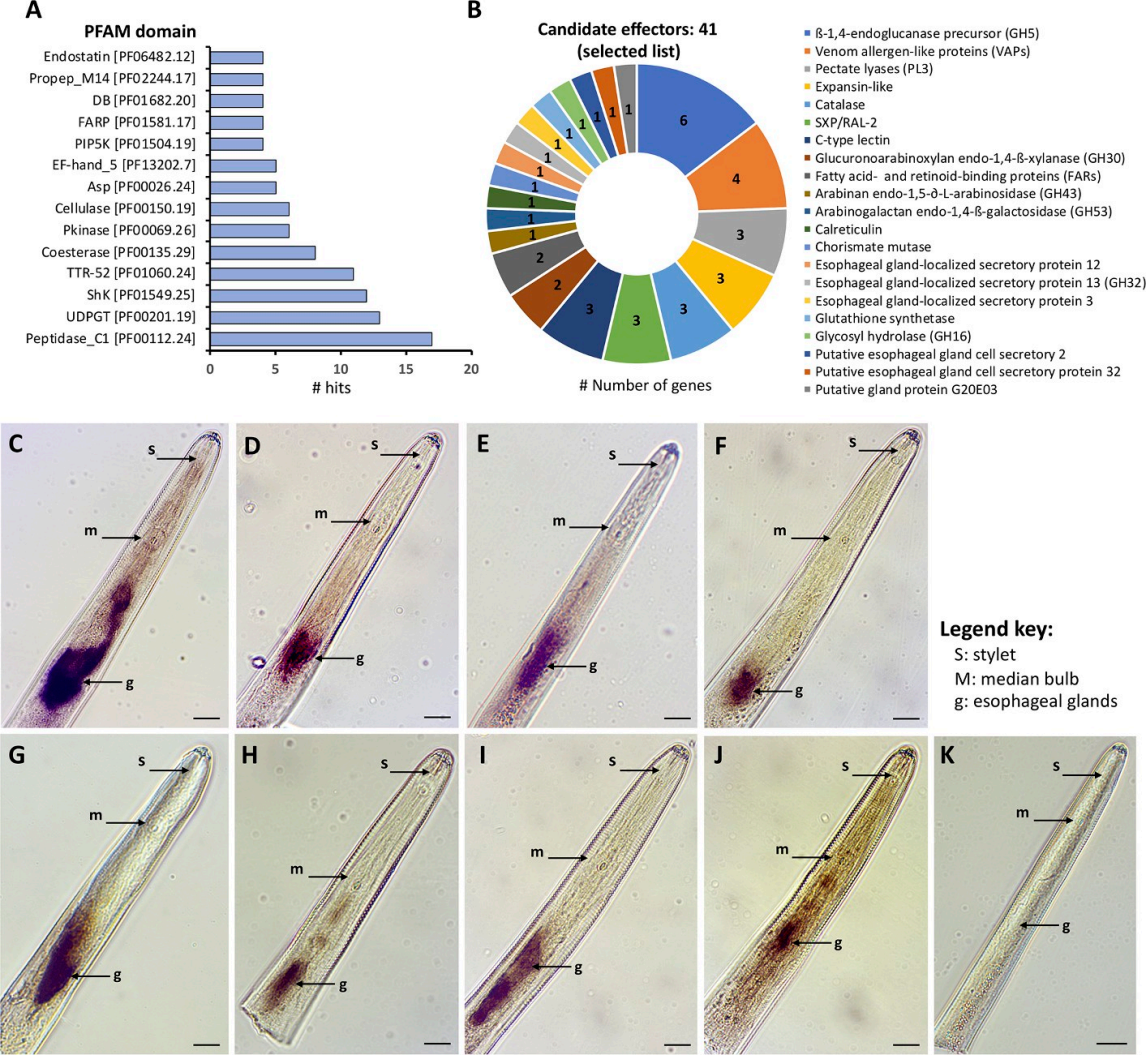
Identification of known candidate effector genes identified in the esophageal gland cell library of *Radopholus similis* by *in silico* analyses. **(A)** Top most abundant PFAM protein domains represented within the list of 542 transcripts coding putative secreted proteins. **(B)** List of selected known candidate effector genes present in the esophageal glands library of *R*. *similis*. **(C-J)** Detection of gene transcripts encoding putative secreted proteins in the esophageal glands of *R*. *similis* by *in situ* hybridization. **(C)** ß-1,4-endoglucanase (positive control, g11661.t1), **(D)** ß-1,4- endoglucanase (g4814.t1), **(E)** pectate lyase (g460.t1), **(F)** expansin-like (g15237.t1), **(G)** venom allergen-like (g14608.t1), **(H)** ShK-Like domain protein (g11934.t1), **(I)** glutathione synthetase (g13505.t1), **(J)** Extracellular solute-binding protein (g10597.t1). (K) Example of a control image obtained using the sense probe (e.g. g460.t1). Bars = 20 μm.

Using the previous annotation, 318 (58.8%) proteins displayed a significant BLAST hit against the nr database (e-value < 10^−5^ and bit score > 50), while PFAM predictions were assigned to 274 (50.7%) sequences (S2 Table). Fig 2A lists the most prevalent PFAM domains within this set of secreted proteins. A detailed examination of the 540 transcripts revealed an extensive overlap with known effectors or parasitism-related genes of other plant-parasitic nematodes (Fig 2B), with some of them being highly abundant in the gland cell library (S2 Table), and therefore, validating the approach. This is well illustrated by the re-identification of several transcripts belonging to different families of CWDEs (Fig 2B). Our mapping results also validated the presence of one GS-like effector within the gland cell library, including additional candidate effectors, such as: catalase, chorismate mutases, C-type lectins, fatty acid- and retinol-binding proteins (FARs), SXP/RAL-2 proteins, venom allergen-like proteins, homologues of several esophageal gland-localized secretory proteins of both cyst and root-knot nematodes (Fig 2B), and a significant number of proteases (S2 Table). Noteworthy is also the presence of transcripts encoding proteins with ShK-like domains (PF01549; Fig 2A). Although proteins containing ShK-like domain(s) are broadly expressed in parasitic and non-parasitic nematodes, these types of proteins have been found as critical components of the excretory/secretory proteome of animal-parasitic nematodes implicated in parasitism [43].

The localization of gene transcripts in the nematode esophageal glands is considered a solid indicator of their potential involvement in parasitism, given the specialized nature of these tissues. In addition to the CWDEs previously localized in the esophageal glands of *R. similis* (i.e., three ß-1,4-endoglucanases [26] and one glucuronoarabinoxylan endo-1,4-ß-xylanase [27]), a total of four other genes with validated gland cell localization were also present within this list. This included a calreticulin [31], cathepsin B and C cysteine proteinases [30,32], and a serine carboxypeptidase [33]. To confirm gland-cell expression among the set of genes with known annotation, *in situ* hybridization assays were performed for 16 additional candidates (S2 Table). While using a previously validated gland cell ß-1,4-endoglucanase (*Rs-eng-2*, [26]) as a positive control (Fig 2C), we were able to confirm gland cell localization for seven additional genes, which included an additional ß-1,4-endoglucanase (Fig 2D), a pectate lyase (Fig 2E), a expansin-like (Fig 2F), a venom allergen-like (Fig 2G), a ShK-domain protein coding gene (Fig 2H), and the GS-like effector (Fig 2I). The gland cell localization of the GS-like effector is noteworthy because all the secreted *R*. *similis* GS-like genes form a separate sub-clade within Clade 2 (Fig 1A). Coupled with the fact that these predicted secreted GS genes are the only GS-like genes with a signal peptide in Clade 2 from any nematode species, suggest that the GS-like genes were neo-functionalized as effectors twice by nematodes, i.e., independently in Clade 2 (by *R*. *similis*) and Clade 3 (by cyst and reniform nematodes). Glutathione synthetase-like genes are apparently fertile genetic capital for effector gene birth. Previously described GS-like effectors are unusual in that they do not carry out a canonical GS function [41]. The functions of these new *R*. *similis* Clade 2 GS-like effectors remain to be determined.

Transcripts of a “bacterial extracellular solute-binding protein” (SBP_bac_6, PF13343) coding gene were also localized in the glands of *R*. *similis* (Fig 2J). This particular gene (g10597.t1) had high identity (BLAST e-value 4e^-114^ and 50% amino acid identity) to a protein of a nitrogen-fixing soil *Rhizobium* bacterium. The presence of introns (n = 5) and transcript coverage in the gland cell library (FPKM = 77) provide further evidence for nematode origin. Putative homologues of this gene were only detected in a few other plant-parasitic nematodes (i.e., cyst, root lesion and false root-knot nematodes), but so far are absent in root-knot nematodes and free-living nematodes. We also found candidate homologues of this gene in the genome assemblies of the soil springtail *Folsomia candida* (Arthropoda, Collembola) and two fungus gnat species, *Bradysia coprophila* and *B*. *odoriphaga* (Arthropoda, Insecta). The latter are important insect pests of plant seedlings due to their damage to the root system [44,45]. Noteworthy is the absence of homologues in remaining metazoan. Our phylogenetic analyses support three possible scenarios (S1 Fig): 1) independent HGT in nematodes and arthropods from bacterial and fungal donors, respectively; 2) one single common origin in the common ancestor of arthropods and nematodes followed by independent gene losses in all other related animals, and 3) convergent evolution in four independent lineages (bacteria, fungi, nematodes and arthropods). To further investigate the likelihood between the first two scenarios, we performed a constrained topology search enforcing the monophyletic grouping of arthropods and nematodes. Although the likelihood was poorer in the constrained tree, it was not significantly poor according to the approximately unbiased (AU) tree topology test. Intuitively, the third scenario seems unlikely, while the first two are equally likely according to our analyses. In either case, the presence of a small family of related genes (n = 8) suggest gene duplication post transfer to *R*. *similis*.

For the remaining genes (n = 8), no signal was observed with the current anti-sense probes, which was also true for all negative control probes of sense orientation (e.g., Fig 2K). Overall, these results suggested that the gland cell library can be used to identify previously known as well as novel effectors.

### The new effector repertoire of *Radopholus similis* is dominated by ‘pioneers’

Many pathogen effectors display rapid evolution and divergence as a consequence of selective pressure from their hosts [46]. Common to other plant-parasitic nematodes [37–40], a significant proportion of the secreted proteome identified in the esophageal gland cells of *R*. *similis* either lack a functional annotation (i.e., no PFAM domain) or detectable homology to other proteins deposited in the nr database at NCBI. Despite our annotation efforts, no function or annotation could be assigned to 202 (37%) transcripts encoding secreted proteins within the gland cell library (S2 Table). Therefore, we used an integrative approach to select a list of effector candidates for further validation. This selection was based on three different assumptions: 1) transcript abundance in the gland cell library, i.e., priority was given to the top 200 most abundant genes (FPKM > 82); 2) lack of annotation (i.e., pioneer genes); and 3) distribution across the phylum through homology BLAST searches. In the last case, priority was given to sequences exclusive to *R*. *similis* or with a significant BLAST hit (e-value < 10^−5^ and bit score > 50) to at least one plant-parasitic nematode, but absent from the animal-parasitic nematode *Brugia malayi* and the free-living nematode *Caenorhabditis elegans*. Through manual assignment, a final list of 60 candidates was selected for further analyses (S2 Table).

**Fig 3:**
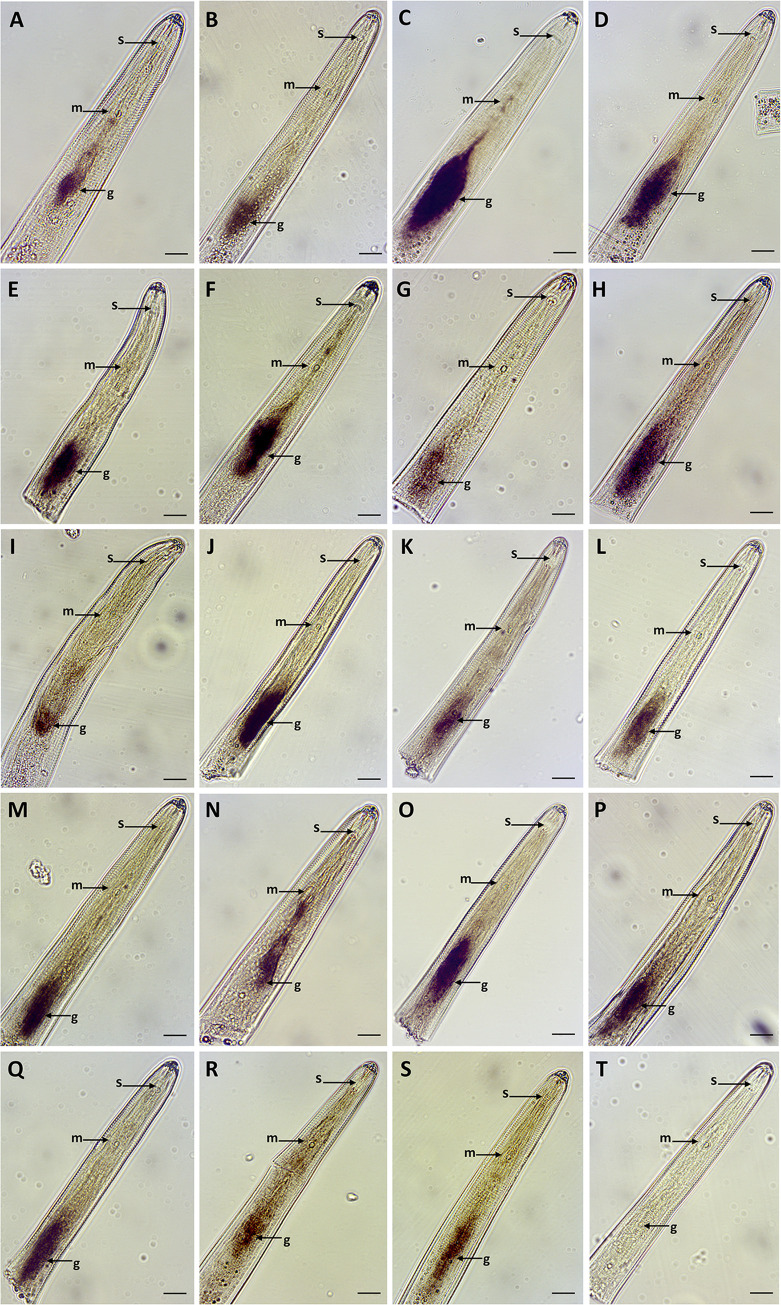
Detection of *Radopholus similis* gene transcripts encoding pioneer secreted proteins by *in situ* hybridization. **(A-S)** Transcripts encoding 19 different genes were localized in the nematode esophageal glands of *Radopholus similis* using the corresponding anti-sense DIG-labeled cDNA probes. **(A)** g9016.t1, **(B)** g5306.t1, **(C)** g14603.t1, **(D)** g10323.t1, **(E)** g2910.t1, **(F)** g10498.t1, **(G)** g4420.t1, **(H)** g1733.t1, **(I)** g8454.t1, **(J)** g3018.t1, **(K)** g14271.t1, **(L)** g13627.t1, **(M)** g2281.t1, **(N)** g11602.t1, **(O)** g7057.t1, **(P)** g4680.t1, **(Q)** g11095.t1, **(R)** g8376.t1, **(S)** g3015.t1. Due to the high variability of the esophageal gland size among different specimens and nematode stages, both dorsal and subventral glands were labeled as esophageal glands. Details regarding each gene abundance within the glands library is presented in S2 Table. **(T)** Example of a control image obtained using the sense probe (e.g. g14603.t1). g: esophageal glands; m: median bulb; s: stylet. Bars = 20 μm.

To validate gland cell expression, *in situ* hybridization assays were performed on a pool of mixed *R*. *similis* developmental stages. mRNA of a total of 19 (out of 60 lacking any functional annotation) genes was specifically localized in the esophageal glands and/or along the esophageal gland cytoplasmic extensions (Fig 3).

**Fig 4:**
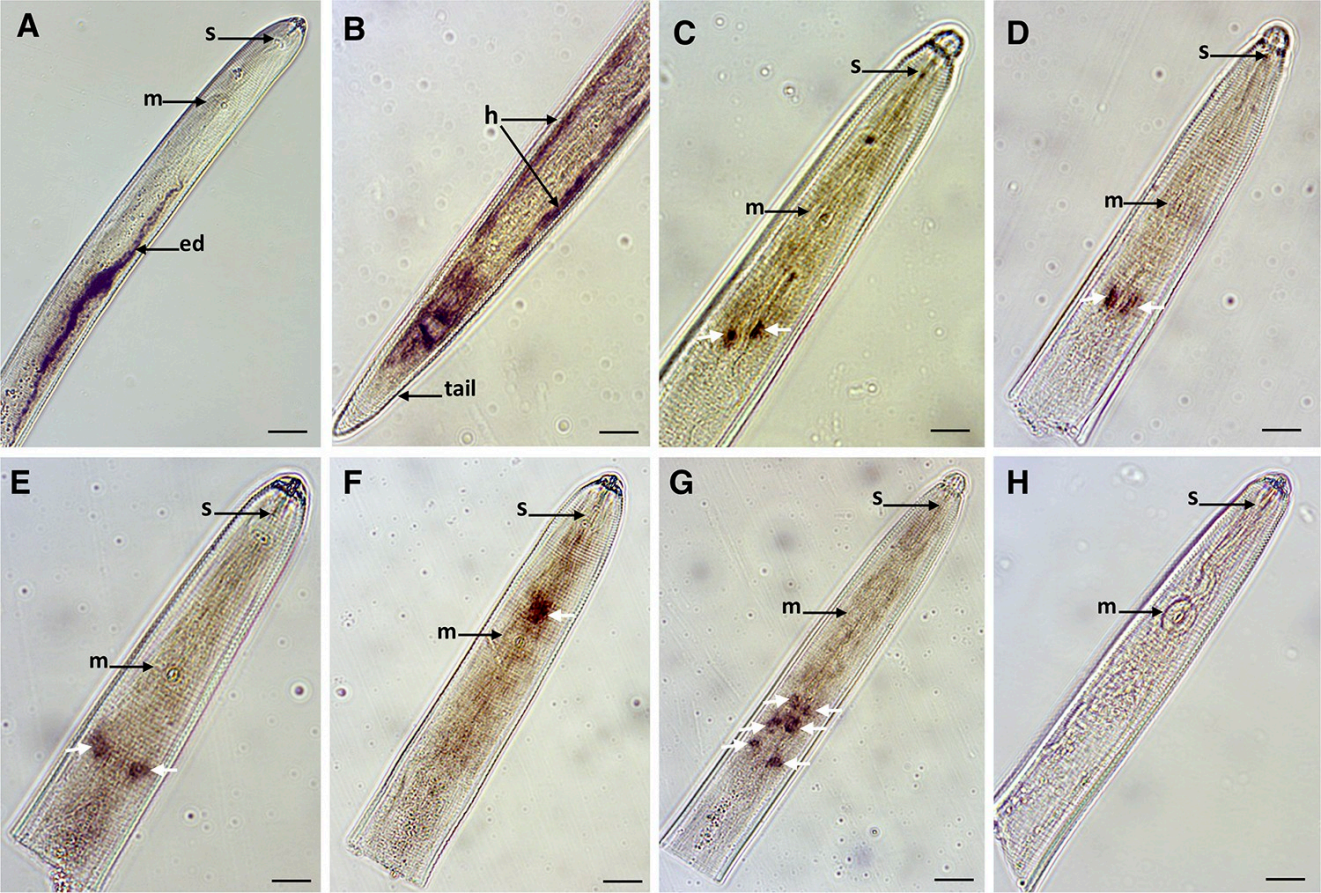
Detection of gene transcripts encoding putative secreted proteins and localized in different *Radopholus similis* tissues by *in situ* hybridization. **(A)** g9088.t1, **(B)** g1157.t1, **(C)** g12860.t1, **(D)** g12895.t1, **(E)** g5235.t1, **(F)** g11460.t1 and **(G)** g13141.t1. White arrows in images C-G indicate “cell-like bodies” along the anterior region of the nematode. **(H)** Example of a control image obtained using the sense probe (e.g. g9088.t1). (ed): excretory duct; m: median bulb; s: stylet. Bars = 20 μm.

This discovery expands the number of validated gland-cell expressed effectors so far identified for *R*. *similis* by 500%, and reflects the wide range of unexplored genes for this species. In addition, transcripts of seven genes were localized in other nematode tissues (Fig 4), some of which may have relevance to parasitism [e.g., the secretory/excretory system (Fig 4A) and in the hypodermis region (Fig 4B)].

Although some genes expressed in the secretory/excretory system and hypodermis have been shown to be relevant for parasitism [47], we cannot exclude that they may be part of the ordinary development or physiology of the nematode, and so further experimentation would be required to classify them as effectors given the nature of these tissues. Transcripts of five other genes were localized in “cell-like bodies” along different parts of the anterior region of the nematode (Fig 4C–4G), which excludes *a priori* their participation as effectors. For the remaining genes (n = 34) no signal was detected using the probes designed in this study, nor with the corresponding sense-probes, which were used as negative controls (e.g., Figs 3T and 4H). Note that considering the number of known effector candidates predicted in the genome, it is plausible that additional effectors are not represented in this gland library.

### Rs-SUG box–a promoter motif associated with SUbventral Gland expression allows the prediction and identification of novel effectors

**Fig 5:**
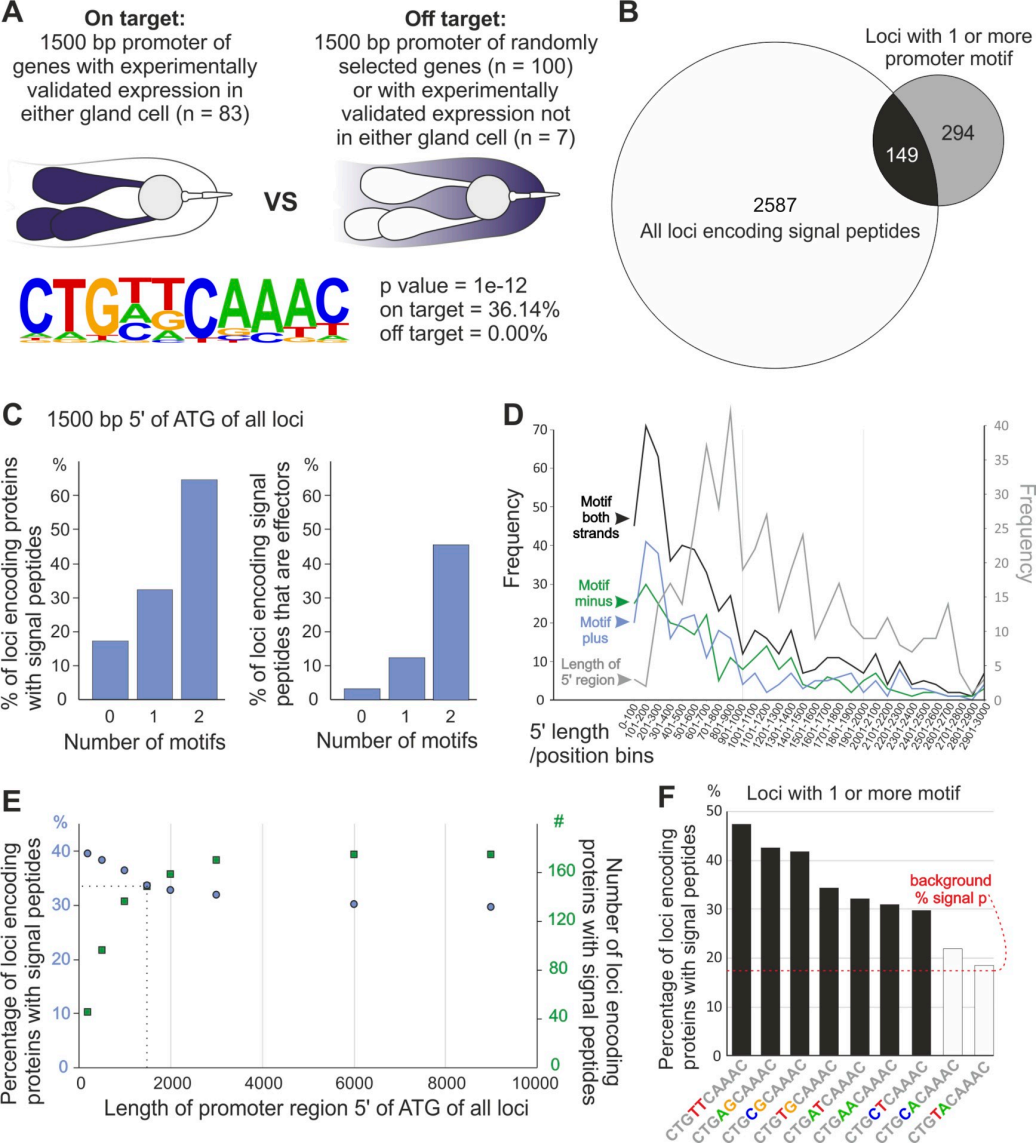
Identification of a non-coding motif associated with subventral gland cell expression in *Radopholus similis*: the Rs-SUG box. **(A)** Graphic representation of the strategy applied to identify a highly enriched consensus motif of 10 nucleotides (CTG[T|A|C][T|G|A]CAAAC) in the promoter region of gland effector genes. **(B)** Number of genes coding predicted secreted proteins containing at least one motif in the promoter region (n = 149). **(C)** The more motifs present in the promoter region, the more likely is that the corresponding gene will contain a signal peptide for secretion. **(D)** The frequency of the motif peaks mainly in the 100–300 bp upstream the start codon and is non-strand specific. **(E)** The distribution of the motif within the 1,500 bp upstream the start codon correlates with genes coding putative secreted proteins. **(F)** Graphic representation of the motif variant combinations. Seven (black bars) out of the nine variants showed enrichment for putatively secreted proteins.

With a more complete, and less biased, roster of *R*. *similis* effectors, we sought to identify potential promoter regulatory elements associated with gland-cell expression. A training set of 83 genes, which included an exhaustive list of homologues of known candidate effectors and genes experimentally validated in the gland cells of *R*. *similis*, was selected (S3 Table). The 1,500 bp promoter regions of these identified candidate effectors were compared to the promoter regions of a negative set [100 randomly selected genes, plus those experimentally validated in other non-gland tissues herein (n = 7)]. Employing a differential motif discovery algorithm, a highly enriched motif was identified (Fig 5A, *p* value = 1e-12). This motif was identified in 36.1% of the training set promoter regions and was completely absent from the negative promoter gene set. The motif of consensus sequence CTG[T|C|A][T|G|A]TCAAAC contains two sites (position four and five) that are variable.

Of the 443 promoters across the entire genome that contained this motif, a third of the corresponding genes (149) encoded a signal peptide for secretion (a hallmark of an effector–Fig 5B). This is a considerable enrichment over the genome as a whole (~14%, *p* value = 1.05e-26). The more copies of the motif in the promoter the more likely the corresponding gene encodes a signal peptide, analogous to other unrelated sequences but conceptually similar effector promoter motifs identified in other plant-parasitic nematode species [40,48,49]. Of those genes with two copies of the motif in their promoter, 60% encode a signal peptide. There were no genes with three copies of the motif in their promoter (Fig 5C). Most, but not all, occurrences of this motif were located within the first 1,500 base pairs (with a peak of occurrence approximately 100–300 bp upstream the coding start site) on the plus and the minus strand (Fig 5D). Due to a combination of this positional enrichment and the average length of promoters in *R*. *similis*, using promoter regions longer than 1,500 bp does identify more secreted proteins but this increase was with diminishing returns and with a reduced overall proportion (i.e., more non-secreted proteins are also included, Fig 5E). Finally, we analyzed each potential two-base pair variant of the motif in positions four and five and determined that two of the nine variants did not enrich for putatively secreted proteins. The final motif consensus is therefore CTGTTCAAAC, where TT at positions four and five is the most common/useful variant, but can be any combination of [T|A|C] and [T|G|A] except CA and TA (Fig 5F). Although we recognize that this pipeline will potentially exclude putative effectors absent of a signal peptide for secretion, our goal was to prioritize our analyses on effector candidates following the classical secretory pathway (which appear to be the dominant majority).

**Fig 6:**
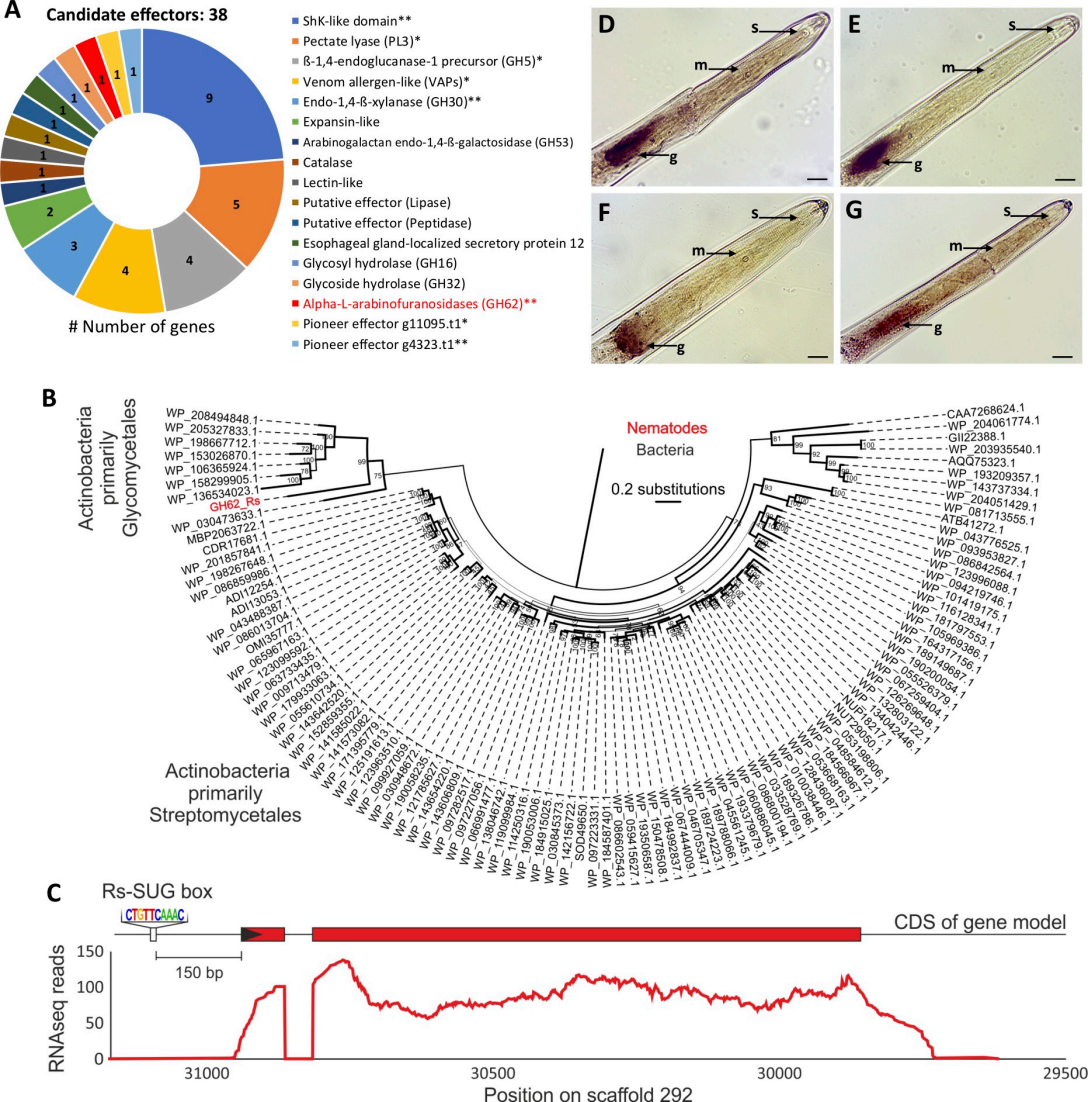
Identification of candidate effector genes through promoter motif searches in the genome of *Radopholus similis*. **(A)** List of candidate effector genes encoding secreted proteins without transmembrane domains identified by promoter motif CTG[T|A|C][T|G|A]CAAAC searches. **(B)** Maximum-likelihood phylogenetic tree of *R*. *similis* GH62 protein sequence (in red) with representative sequences retrieved from bacteria at NCBI (100 top hits). Bootstrap values are presented in the corresponding branches. **(C)** Graphic representation of *R*. *similis* GH62 gene model and corresponding RNAseq coverage. Exons are represented by red boxes, and signal peptide by a black arrow. **(D-G)** Genes associated with the Rs-SUB box coding predictive secreted proteins were localized in the nematode esophageal glands by *in situ* hybridization: **(D)** α-L-arabinofuranosidase GH62 (g448.t1), **(E)** endo-1,4-ß-xylanase GH30 (g15208.t1), **(F)** ShK-domain protein (g6688.t1), **(G)** pioneer gene (g4323.t1). *Validated in the esophageal glands in Fig 2. **Validated in the esophageal glands after the Rs-SUB box identification. g: esophageal glands; m: median bulb; s: stylet. Bars = 20 μm.

To determine whether this motif was preferentially associated with effector expression in the dorsal or the subventral glands, we analyzed genes that contained one or more motif in the 1,500 bp upstream of the coding start site and that encoded putatively secreted proteins (i.e., presence of a signal peptide and absence of transmembrane domain/s). This list contained 109 genes, many of which have a long body of literature unanimously supporting their expression in the subventral glands of a range of other closely related plant-parasitic nematodes (e.g., different CWDEs, VAPs), including those without previous representation in the gland cell library (Fig 6A and S4 Table). Importantly, of those genes in this study that show a clearly distinguishable signal, they are clearly labelling the subventral glands. Taken together, we concluded that this motif is specifically associated with the subventral glands, the first sub-ventral gland-specific motif for any plant-parasitic nematode, and named it the *R*. *similis* SUbventral Gland Box (Rs-SUG box). The identification of putative *cis-*regulatory elements could help in the identification of transcription factor(s) responsible for the modulation of the associated effectors [18].

To determine whether the Rs-SUG box can be used to identify new effectors *a priori*, several novel sequences that encode a signal peptide but were not previously characterized as effectors were also identified. These include a gene coding a secreted α-L-arabinofuranosidase belonging to the family GH62 (PFAM PF03664, e-value 1.7e^-55^). Members of this family are considered to be phylogenetically restricted to bacteria and fungus/oomycetes [50] and were involved in plant penetration and pathogenesis of fungi [51,52]. To the best of our knowledge, this is the first report of a GH62 member in any metazoan. The domain structure of the nematode GH62 protein was highly similar to modern day bacteria (70% identity across 286 amino acids, S2 Fig). Phylogenetic analyses support the horizontal transfer of this gene from bacteria with a most closely related modern-day decedents of the donor in available sequence databases belonging to *Glycomyces* and *Streptomyces* bacteria (Actinobacteria, Fig 6B). Species of both genera have been found as part of the root endophytome of banana [53], one of the many hosts of *R*. *similis*. Interestingly, Actinobacteria close relatives are often associated as potential donors of HGT acquired by other plant-parasitism nematodes [29,54]. To validate the nematode origin of this gene, both full-length genomic and cDNA sequences were cloned from the Hawaii population, revealing the presence of one intron. Remarkably, the first exon encodes only 25 amino acids, 24 of which were the signal peptide. The second exon was unusually long and encoded the rest of the protein (318 amino acids) as a single open reading frame. Despite the unusual genetic structure, we have ample RNA-seq transcript coverage support (Fig 6C). The absence of similar GH62 sequences in any other metazoan (including closely related plant-parasites), the unusually high sequence similarity to bacteria, and the unusually low number of introns in the nematode sequence, suggest that this is a rare example of an extraordinarily recent HGT event. While recent, it certainly pre-dates the globalization of *R*. *simili*s because a survey of draft genomes of eleven geographically isolated populations of *R*. *similis* (from Africa, Australia, the Americas, and Hawaii—Wram et al. pers. com.) confirmed the presence of a single GH62 in each, with identical gene structures (S3 Fig). It is generally accepted that after HGT events from prokaryote species, genes acquire intron(s), signal peptides, and promoters in order to be utilized by the recipient genome as effectors. While it is tempting to speculate that the single short intron, the short exon that almost exclusively encodes a signal peptide, and the Rs-SUG box-containing promoter were transferred as a unit, their sequences are unlike any other in the nematode genome: it is not clear where they came from.

In addition to the GH62, this set of putative secreted proteins associated with the motif also contained 40 genes (36% of the total set) that had no recognizable functional domains or BLAST hit against the nr dataset, suggesting the possibility of finding additional effectors yet to be discovered for *R*. *similis* and unlike effectors in other plant-parasitic nematodes (S4 Table). To provide further evidence of the promoter motif prediction, 10 additional genes were chosen for *in situ* hybridization, including the new GH62 found for *R*. *similis*. Transcripts of four genes were validated within the esophageal glands, which included the GH62 gene (Fig 6D), one xylanase (Fig 6E), one protein with ShK-like domain (Fig 6F) and one pioneer (Fig 6G). Overall, the identification of the Rs-SUG box is a valuable and complementary additional criterion to expedite genome-wide effector prediction *de novo*, unbiased by previous efforts in related but nevertheless quite different species. Considering that a significant number of genes represented by this motif encode secreted proteins, it stands to reason that additional effectors may also be present among these candidates.

### Pioneers are the most highly expressed effectors during parasitism

**Fig 7:**
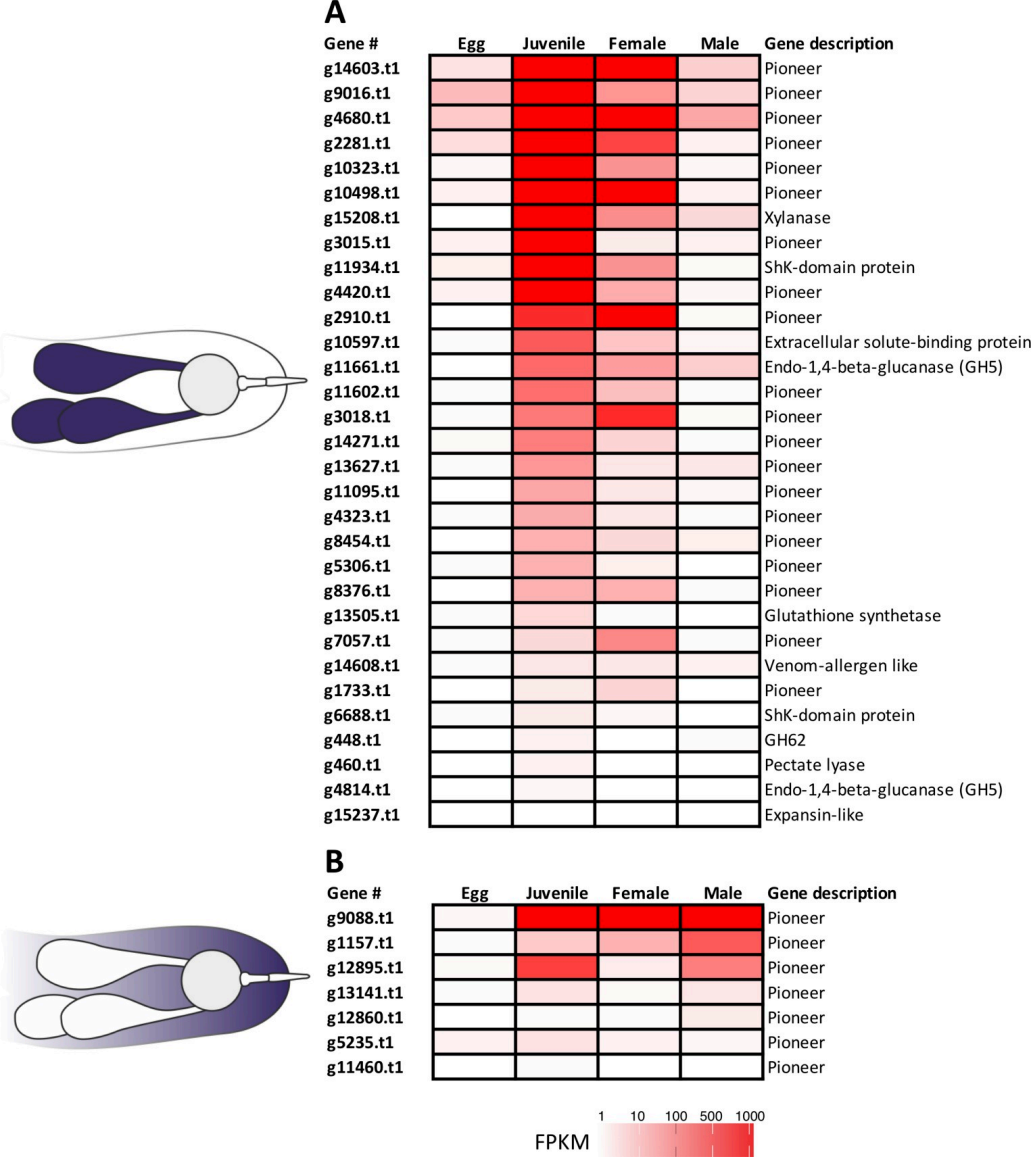
Gene expression analyses of candidate effector genes of *Radopholus similis* across different nematode developmental stages. **(A)** Genes validated in the esophageal glands of the nematode as shown in Fig 2. **(B)** Genes validated in different nematode tissues as shown in Fig 3. Heat maps represent the expression levels calculated using the fragments per kilo base of transcript per million mapped reads (FPKM) of specific nematode developmental stages RNA-seq libraries (Bioproject PRJNA427497).

To obtain insight into the expression profiles of the new effector set throughout the nematode life cycle, we took advantage of available public transcriptome RNA-seq datasets (Bioproject PRJNA427497 [23]) generated for different nematode developmental stages (i.e., eggs, juveniles, females and males) of *R*. *similis* and generated additional RNA-seq datasets for discrete times after infection. In general, juveniles and females had higher relative expression of effectors compared to other developmental stages (Fig 7A). This was in stark contrast to males and, even more so, eggs where low or even no gene expression was detected for most effectors. This dynamic pattern of expression correlated well with the migratory and biological status of both juveniles and females within the roots. While juveniles have a more dynamic migratory activity, females can be more static by spending significant periods of time feeding and laying eggs, which could relate to the generalized higher abundance of the full range of effectors in juveniles. Although males are able to migrate, they do not feed and they have reduced esophageal glands [8], which could reflect the lower expression found for the majority of these genes. As nematode eggshells can contain both J1 and J2, it is likely that the detection of some effectors in the eggs library could correspond to the expression of these genes in J2 nematodes within eggs just prior to hatching (Fig 7A).

In contrast, transcripts localized in other nematode tissues (e.g., E/S system, hypodermis or “cell-like bodies”) were more evenly expressed across all migratory stages (Fig 7B), suggesting that these genes might be involved in fundamental molecular functions of the nematode and not parasitism.

**Fig 8:**
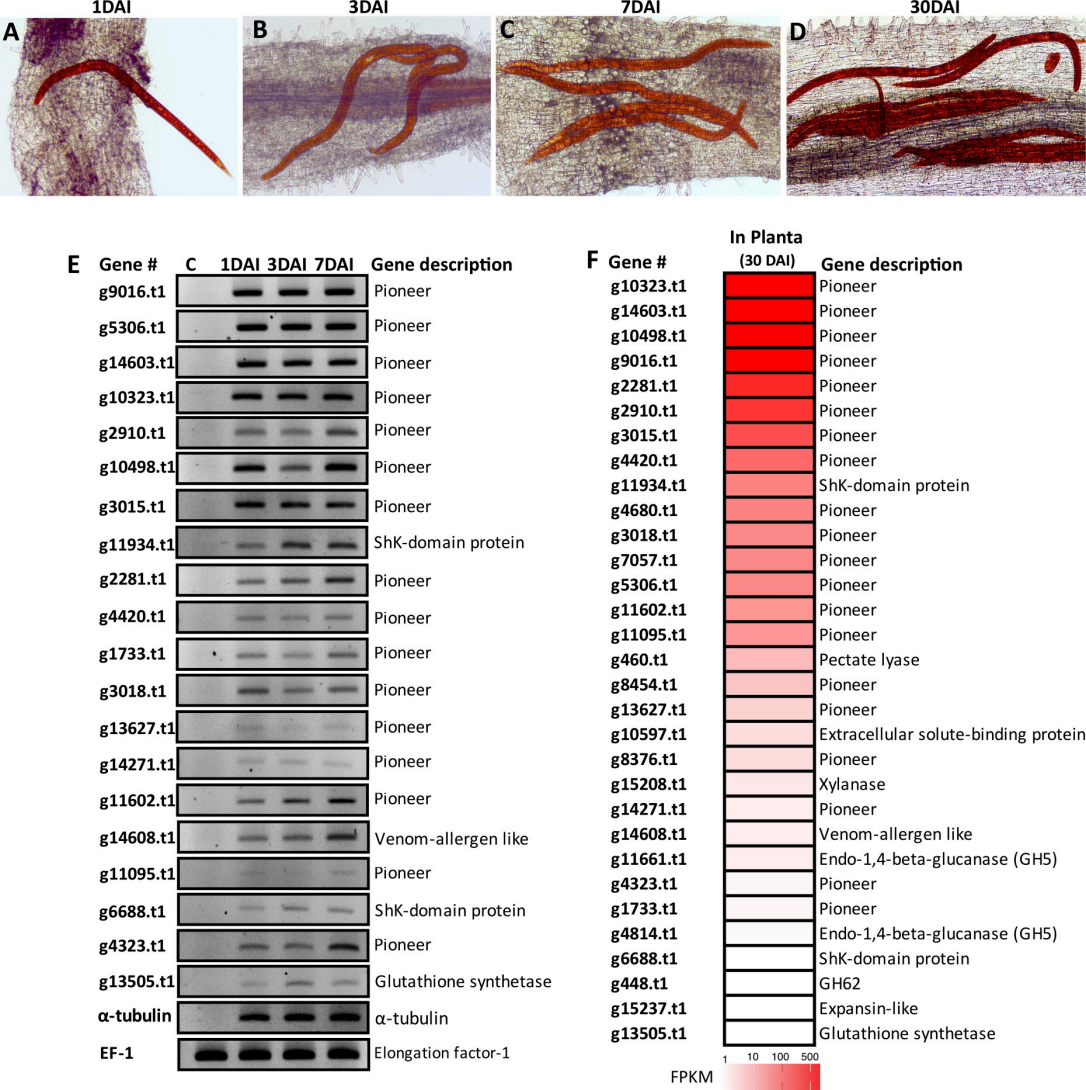
Expression profile of *Radopholus similis* candidate effector genes in *planta*. **(A-D)** Acid fuschin staining of nematodes (red color) in carrot hairy roots at 1 **(A)**, 3 **(B)**, 7 **(C)** and 30 **(D)** days after inoculation. **(E)** Semi-quantitative RT-PCR validating the expression of 20 candidate effectors in carrot hairy roots at 1, 3 and 7 DAI. As a positive control, all cDNA libraries were amplified with primers derived from the *α*-tubulin gene of *R*. *similis* or the translation elongation factor EF-1 alpha gene (*EF-1α*) of carrot. C corresponds to non-infected carrot hairy roots. **(F)** Heat map representing the expression profile of 30 candidate effectors validated herein in the esophageal glands of *R*. *similis*. The expression levels were calculated using the fragment per kilobase of transcript per million mapped reads (FPKM) values of two independent RNA-seq libraries generated from mixed nematode stages collected from carrot hairy roots at 30DAI.

To understand the expression of effectors over time, carrot hairy roots were inoculated with mixed nematode stages and assessed at 1, 3, 7 and 30 days after inoculation (DAI). At 1 DAI, individual nematodes were found penetrating into the roots (Fig 8A). At 3 DAI, some nematodes were fully established in the cortex cells of the roots (Fig 8B), while at 7 DAI an increasing number of nematodes were observed within the inner layers of the roots (Fig 8C). Thirty DAI agglomerates of nematodes were found along different areas of the root (Fig 8D) and associated with an increased number of necrotic root tissue.

Semi-quantitative RT-PCR analyses were then performed for 20 new effectors, with particular emphasis on pioneer genes at 1, 3 and 7 DAI (Fig 8E). All candidate effectors were detected at the three time points but at varying levels of expression. Additionally, RNA sequencing of two independent nematode samples extracted from well-established carrot hairy roots at 30 DAI showed that among the effectors, the pioneers dominated the most highly abundant effector transcripts of nematodes in roots (Fig 8F). There are multiple lines of evidence that several unrelated pioneer effectors of both cyst and root-knot nematodes have potential for host defense suppression [55,56]. Functional analyses of these pioneer effectors of *R*. *similis* are of particular interest to confirm and identify their role(s) in parasitism.

### A platform for functional characterization of *Radopholus similis* effectors

**Fig 9:**
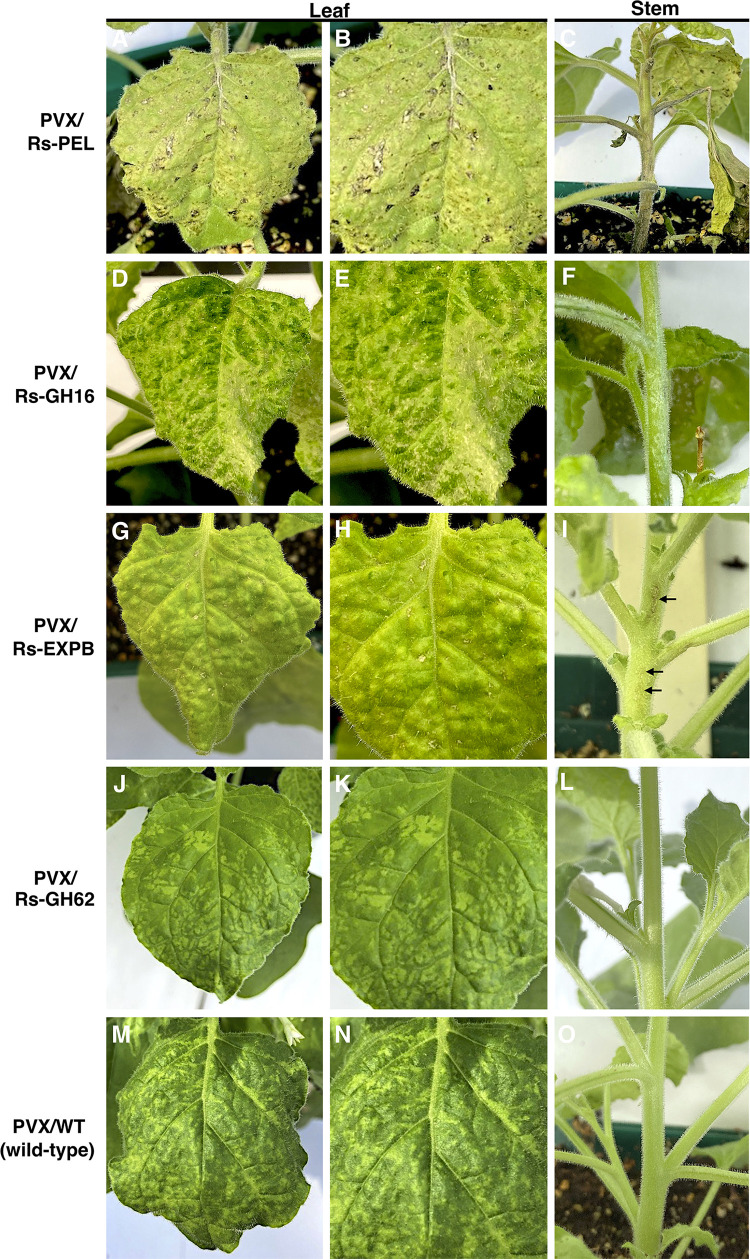
Phenotypic changes in *Nicotiana benthamiana* plants infected with recombinant potato virus X carrying cell wall-modifying genes of *Radopholus similis*. **(A-C)** Expression of Rs-PEL gene induced large necrotic lesion and overall chlorosis in leaves **(A-B)** and stems **(C)** of the infected plants. **(D-F)** Expression of Rs-GH16 gene induced chlorotic and uneven appearance of the leaf tissue, as well as necrotic lesion in the leaves (D-E), and pitting or grooving-like phenotype in the stems ((indicated by arrows in F). **(G-I)** Plants expressing PVX/Pp-EXPB displayed necrotic spots in the leaves (G-H), and lesion-like spots in the stems (indicated by arrows in I). **(J-L)** Typical mosaic-like symptoms induced by PVX/RsGH62 and **(M-N)** PVX wild-type (PVX/WT) on leaves of *N*. *benthamiana* plants. All photos were taken 14 days after inoculation.

Functional characterization of plant-parasitic nematode effectors is the next bottle neck after identification [18]. Given the advances in effector identification for *R*. *similis*, we also sought to expedite effector characterization in this species. We selected four CWDEs that were likely acquired by HGT (the new GH62, a GH16, a pectate lyase, and an expansin-like gene) for experimental validation. This was accomplished by evaluating their ability to elicit phenotypic responses in a model plant *Nicotiana benthamiana* via transient expression assays using a PVX-based vector. The full-length coding sequence of each candidate (including the signal peptide because CWDEs are often secreted by plant-parasitic nematodes to the apoplasm during invasion and migration of the host roots [19]) was cloned into a PVX-based vector (pP2C2S) [57]. Symptoms on *N*. *benthamiana* plants and phenotypic differences were registered at 14 and 21 DAI (Fig 9).

The most distinct phenotype occurred in plants inoculated with the PVX/Rs-PEL construct, where large necrotic lesions and chlorosis were observed on the leaves and stems at 14 DAI (Fig 9A–9C), and by 21 DAI plants exhibited a strong necrotic reaction and general decline in health (S4A Fig), including the root system (S4B Fig). Inoculation with the PVX/Rs-GH16 construct resulted in chlorotic and an uneven appearance of the leaf tissue, as well as vein necrosis and necrotic lesions (Fig 9D and 9E). The stems of PVX/Rs-GH16-infected plants displayed pitting or a grooving-like phenotype (Fig 9F). Plants inoculated with PVX/Rs-EXPB exhibited small necrotic lesions on leaves and uneven appearance of the leaf tissue (Fig 9G and 9H) along with noticeable stem necrosis (Fig 9I). Plants expressing PVX/Rs-GH62 displayed typical mosaic symptoms with dark light-green patches (Fig 9J–9L), similar to the phenotype induced by PVX/WT (Fig 9M–9O). Expression of all PVX constructs in *planta* was confirmed by RT-PCR (S5 Fig), followed by the sequencing of the PCR products. The plant phenotype observed (with the exception of GH62) was coherent with a putative activity of these proteins in tissue maceration and breaking down of the plant cell-wall components during penetration and migration of *R*. *similis*. Nevertheless, we cannot exclude that some of these genes may be recognized by the host, triggering a plant immune response. We therefore demonstrated both a new way to identify candidate effectors (Rs-SUG box) but also an expeditious way to test their function in *R*. *similis* using a high throughput assay. Taken together, these results may pave the way for substantial advances in effector biology for *R*. *similis*.

### An evolutionary view of effector gene birth and death in *Radopholus similis*

**Fig 10:**
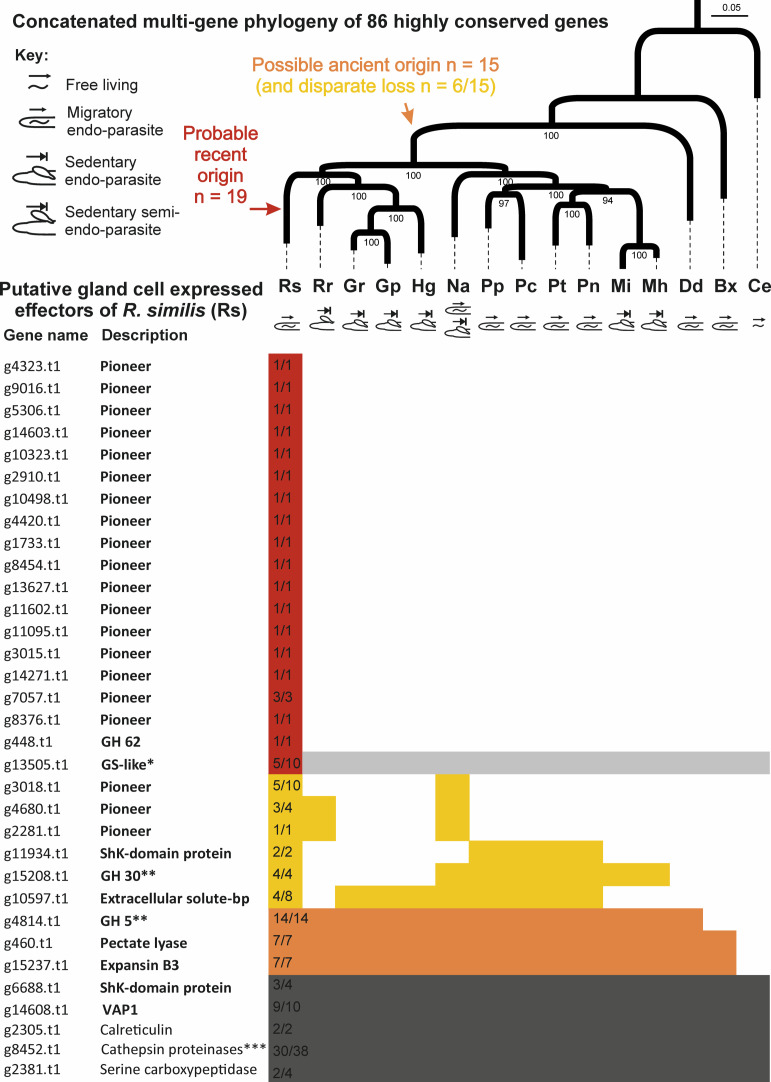
*Radopholus similis* holds a diverse and emergent repertoire of effectors shaped by diverse evolutionary events. Top panel corresponds to a schematic phylogeny of the phylum Nematoda based on 86 highly conserved genes among plant-parasitic nematodes with distinct lifestyles, and the free-living nematode *Caenorhabditis elegans* (adapted from [38]). The lower panel indicates the presence/absence of putative homologues through BLAST analyses (e-value < 10^−5^ and bit score > 50) using as query the full set of effectors identified so far for *R*. *similis*. The new candidate effectors identified in this study are represented in bold (n = 30). Numbers within the cells of *R*. *similis* correspond to the number of genes coding putative secreted proteins in relation to the total number of related genes found in the Rv genome. *Both GH30 and GH5 are represented by the corresponding genes (g15208.t1 and g4814.t1) validated in this study, while cathepsin proteinases are represented by g8452.t1. Legends: *Pratylenchus penetrans;* Pc: *Pratylenchus coffeae*; Pn: *Pratylenchus neglectus*; Pt: *Pratylenchus thornei*; Mi: *Meloidogyne incognita*; Mh: *Meloidogyne hapla*; Na: *Nacobbus aberrans*; Rs: *Radopholus similis*; Rr: *Rotylenchulus reniformis*; Hg: *Heterodera glycines*; Gr: *Globodera rostochiensis*; Gp: *Globodera pallida*; Dd: *Ditylenchus destructor*; Bx: *Bursaphelenchus xylophilus;* Ce: *Caenorhabditis elegans*. RKN: root-knot nematodes; CN: cyst nematodes.

Combined, we validated 30 additional effector genes in the glands (5-fold more than all previous publications on the subject combined). This effort drastically expanded effector repertoire in the context of the evolution of plant-parasitism by nematodes. We therefore mapped the presence or absence of *R*. *similis* effectors in other nematodes to a robust phylogenetic scaffold of these species reconstructed using a multi-gene phylogeny based on 86 CEGMA (Core Eukaryotic Genes Mapping Approach) genes [38]. The topology of the species tree supports the phylogenetic proximity between *R*. *similis* and cyst nematodes (as opposed to the Pratylenchidae, Fig 10), as previously shown using different molecular markers [12,15], and using similar phylogenomic analyses, such as the concatenation of widely conserved low-copy number of proteins [16].

Based on our current resources, these analyses revealed a striking pattern of evolution. Broadly speaking there are two classes of effectors: the ones widely conserved in plant-parasitic nematodes and evolutionarily ancient and the lineage-specific new ones. The majority of the pioneer effectors (i.e., genes without a predictive function; n = 17/20) are specific to *R*. *similis*, lacking recognizable homology in all the other nematodes investigated here. Hence, emergence of a substantial set of effectors occurred since the divergence from the last common ancestor in the phylogeny and is illustrative of how little we knew about the peculiarities of *R*. *similis*. Prior to this study, there was not a single known pioneer effector for *R*. *similis*, and yet, our effort demonstrates that half of the effectors discovered in *R*. *similis* are pioneer. Assuming the *Radopholus*-specific genes have not been simply overlooked in the other genomes, several hypotheses may explain the presence of such species-specific orphan genes: (i) HGT of non-Nematoda origin specific to the *Radopholus* lineage; (ii) duplication followed by extreme divergence [58]; and/or (iii) ‘true’ *de novo* gene birth from non-coding sequences in the genome [59].

Many of these pioneers are apparently singletons (i.e., no similarity to another gene in *R*. *similis*), potentially supporting a more recent origin. Other effectors that can be categorized as very new include the recent horizontal transfer event (i.e., GH62) and the independent neofunctionalization of GS-like effectors. On the other hand, all other effectors had some evidence of an older origin, i.e., before the bifurcation in the tree that separates *R*. *similis* from the root-knot nematodes, because they had various distributions of similar hits on both sides of the split. These included the remaining three pioneers (g3018.t1, g4680.t1, g2281.t1) with significant tBLASTn hits to the semi-endoparasitic nematodes *Rotylenchulus reniformis* and/or *Nacobbus aberrans* sequences (Fig 10). It is noteworthy that these three species are predominately distributed in tropical, subtropical and warm temperate zones and share signatures of both migratory and sedentary plant-parasitic nematodes [12,60–62]. Similarly, all effectors with putative functional annotations are shared by many *taxa* across the phylogeny (including in some cases the free-living nematode *C*. *elegans*) and some show various patterns of disparate loss across the phylogeny. Those with representatives in both free-living and plant-parasitic nematodes likely represent further evidence for gene duplication followed by neofunctionalization and co-option for effector functions (e.g., GS-like effectors). The fact that we have very old and the very new effectors, and have identified nothing in-between (i.e., conserved effector genes with *R*. *similis* and either reniform or cyst but not the others) does not mean they do not exist, only that they are rarer than the other categories described. An explanation for this observation would be the absence of sequence information for sufficiently closely related species to *R*. *similis*. It will be important to improve the resolution of genomic resources for the plant-parasitic nematodes of Clade 12 to address this observation more concretely (e.g., *Dolichodorus* and *Belonolaimus* spp. would be useful [63]). In addition, these resources may permit the identification of yet more novelties (c.f. first GH62 in a metazoan in *R*. *similis*), broadening our understanding of HGT and plant-parasitism at the same time.

Our analyses support the long-standing idea that HGT is also a major source for the acquisition of new genes in *R*. *similis*, most likely transferred from multiple ancestral bacterial lineages, in line with most CWDEs so far reported for other plant-parasitic species of Clade 12 [28,29]. Importantly, we use effector promoter motif analysis to add to the literature on HGT events: documenting the first example, and a very recent one, of a GH62 coding gene for the phylum Nematoda, and the first time ever reported for a metazoan. The presence of this gene in *R*. *similis* could reflect its adaptation process to specific hosts and/or related environments. Likewise, previously we identified the first pectin methylesterase gene for the phylum Nematoda in *P*. *penetrans*, which is also a migratory nematode [64]. The configuration of CWDEs and pioneer effectors restricted to migratory nematodes indicate a more intricate arsenal of effectors than previously thought, thus, highlighting the need of more comprehensive molecular studies of this group of nematodes. Sequencing of additional species within this genus and closer taxa, coupled with less biased effector prediction methods shown here in (i.e., gland cell sequencing and effector promoter prediction) will refine our understanding of the adaptations to parasitism by migratory nematodes.

## Conclusions

In this study by combining comparative genomics and transcriptomics, targeted gland cell sequencing, and effector promoter prediction, we have dramatically expanded the catalogue of effectors for *R*. *similis*. Analysis of gene contents revealed that *R*. *similis* holds a diverse and emergent repertoire of effectors, which has been shaped by various evolutionary events (including neofunctionalization, HGT, and possibly by *de novo* gene birth) and largely overlooked by a historically sedentary endoparasite-centric stance. Before this study, not a single pioneer effector was known for *R*. *similis*, and yet such pioneer effectors now dominate (in number and expression in *planta*). We searched for homologous across different plant-parasitic nematodes and found that the majority of these predicted effectors are specific to *R*. *similis*. The identification of the Rs-SUG box associated with the promoter region of a significant number of effectors suggests a coordinated expression modulation of these genes and proves a useful tool for effector prediction (including revealing never before seen classes of glycosyl hydrolases in metazoan). Future efforts should focus on the identification and characterization of the host targets of these effectors to determine their biological roles and, thus, help to develop crops with durable resistance this nematode.

## Material and methods

### Nematode collection

*Radopholus similis* originally collected from a commercial *Anthurium andraeanum* farm in Hilo (Hawaii, USA) was used in all experiments. Nematodes were cultured and kept under sterilized conditions on carrot (*Datura carota*) discs at 25°C. Store bought carrots were washed with water and surface disinfested by soaking in a 10% Chlorox solution for 5 min. Carrots were then flamed in a laminar flow hood, peeled, and sliced transversely (10 mm thick, 30–40 mm diameter). Individual carrot discs were then placed in a sterile petri dish without media, and inoculated with nematodes [65]. Nematodes were re-cultured every month onto new carrot disks and maintained in the dark at 25°C.

### *Radopholus similis* secretome predictions

The genome of *R*. *similis* previously generated for the Rv population originally collected from Costa Rica was used as input for the following analyses [24]. The full set of 14,817 protein variants were initially submitted to SignalP 4.0 for prediction of secreted proteins [66], and transmembrane domains (TMHMM) using the TMHMM server version 2.0 (http://www.cbs.dtu.dk/services/TMHMM/). All proteins bearing a signal peptide for secretion and no transmembrane domain were considered putatively secreted. Putative secreted proteins were then annotated using PFAM domain searches against the PFAM-A v33 dataset obtained at https://pfam.xfam.org [67] and run through CLC Main Workbench v.7. BLASTp analyses were performed against the NCBI non-redundant (nr) protein sequence database (e-value < 1e^-5^ and bit score > 50). BLAST2GO [68] was used with default parameters to perform InterProScan annotation. CAZyme annotations for all predictive proteins were determined using the dbCAN2 meta server ([69]; accessed on Jan. 15, 2021). Proteins with consistent carbohydrate-active enzymes (CAZyme) predictions from at least two of the three dbCAN search methods were selected as putative CAZymes for subsequent analyses. Nematode secreted proteins with a putative role in parasitism were identified by the presence of particular domains (PFAM searches) with significant threshold (e-value < 1e^-5^), or by using individual sequences of known nematode effectors [25] as query from the literature covering genes with gland cell localization from different plant-parasitic nematodes (e-value < 1e^-5^ and bit score > 50).

### *Radopholus similis* gene selection

To select a more restricted panel of candidate genes a set of ‘454’ pyrosequencing reads derived from mRNA collected from the esophageal glands of *R*. *similis* [42] were mapped against the 14,817 transcripts predicted in the genome of the Rv population [24]. These reads were mapped using minimap2 with default parameters. RNA read counts were TMM normalized and a FPKM (Fragments Per Kilobase of transcript per Million mapped reads) matrix was generated using Trinity (v2.8.4—run_TMM_normalization_write_FPKM_matrix.pl). Transcripts represented in the gland cell library were additionally compared using BLASTx and tBLASTx (e-value cutoff of < 1e^-5^ and bitscore > 50) to a set of genomes/transcriptomes of plant-parasitic nematodes publicly available at NCBI or Wormbase (http://parasite.wormbase.org). This set was comprised of sequenced genomes/transcriptomes of nematodes distributed by the following phylogenetic clades [63]: 1) Clade 12B: root-knot nematodes *Meloidogyne incognita* [70] and *M*. *hapla* [71]; cyst nematodes *Globodera pallida* [35], *G*. *rostochiensis* [48], and *Heterodera glycines* [72]; false root-knot nematode *N*. *aberrans* [60]; the reniform nematode *R*. *reniformis* [73]; *Pratylenchus coffeae* [74], *P*. *penetrans* [75], *P*. *neglectus* and *P*. *thornei* [38]; 2) Clade 12A: *Ditylenchus destructor* [76]; 3) Clade 10: *B*. *xylophilus* [77]; 4) Clade 9A: free-living species *C*. *elegans* [78]; and clade 8B) the animal-parasitic nematode *Brugia malayi* [79]. A final set of 60 candidates were manually selected within the top 200 most represented transcripts.

### Nematode RNA extraction and cDNA libraries

Total RNA was extracted from a pool of mixed stages of *R*. *similis* (eggs, juveniles and adults) using the RNeasy Plant Mini kit (QIAGEN) according to the manufacturer’s instructions. RNA was treated with RNase-free DNase (QIAGEN) before reverse transcription. The quantity and quality of the extracted RNA was assessed by a ND-1000 NanoDrop spectrophotometer (Thermo Scientific, Waltham, MA, USA) and cDNA was synthesized using the iScript first-strand synthesis kit (Bio-Rad, Hercules, CA, USA) following the manufacturer’s instructions.

### *In situ* hybridization assays

Whole mount *in situ* hybridizations were performed using mixed stages of *R*. *similis* following the protocol of [80]. Specific primers were designed to amplify a range of products (143 to 335 bp) for the candidates using a cDNA library produced from a pool of *R*. *similis* stages (S5 Table). The resulting PCR products were then used as a template for generation of sense and antisense DIG-labeled probes using a DIG-nucleotide labelling kit (Roche, Indianapolis, IN, USA). Hybridized probes within the nematode tissues were detected using an anti-DIG antibody conjugated to alkaline phosphatase and its substrate. Nematode segments were observed using a Nikon Eclipse 5*i* light microscope (Minato City, Tokyo, Japan).

### Promoter analyses

Promoter motif enrichment analyses were carried out as previously described for cyst and root knot nematodes [48,49]. In brief, the 1500 bp promoter regions of 83 putative homologues of genes with validated expression in either gland cell of plant-parasitic nematodes [including 27 validated in *R*. *similis* herein, and 56 validated in other species with sequence similarity to genes in *R*. *similis* (BLAST, e-value < e^-5^) and encode a signal peptide] were compared to the promoter regions of genes unlikely to be expressed in the gland cells (including 7 validated in *R*. *similis* to be expressed in other tissues herein, and 100 other genes randomly selected from the genome). Enriched motifs in the effector promoters were identified using the differential motif discovery algorithm HOMER [81]. Occurrences/locations of motifs of interest (or variants thereof) were identified using the FIMO server [82].

### Plant inoculation and effector gene expression

To follow the early steps of nematode infection, carrot hairy roots were inoculated with nematodes and evaluated at 1, 3, 7 and 30 days after inoculation (DAI). Nematode infected roots were stained with acid fuchsin following the protocol described by [83]. Root tissues were then distained using a clearing solution (equal volumes of lactic acid, glycerol, and distilled water) for 2 to 4 hr at room temperature. After rinsing several times with tap water, roots containing nematodes were stored in acidified glycerol (five drops of 1.0 M HCl in 50 ml of glycerol) and observed using a Nikon Eclipse 50i light microscope.

Semi-quantitative RT-PCR analyses were then performed using total RNA extracted from nematode-infected carrot hairy roots collected at 1, 3 and 7 DAI, using the same methodologies as described above. The same set of primers selected for *in situ* hybridization were used for nematode transcript amplification. The following genes were used as references: the *α*-tubulin gene for *R*. *similis* [84], while the translation elongation factor EF-1 alpha gene (*EF-1α*) of carrot was used as host reference gene [85]. Two independent RNA-seq libraries were generated from a pool of nematode stages collected from carrot roots (30 DAI). Total nematode RNA extraction was carried out as described above. RNA samples having absorbance ratios of 260:280 nm and 260:230 nm above 2, and an integrity value (RIN) above 9 were used for sequencing using the service provided by Novogene (Sacramento, CA, USA). The sequencing libraries were generated using a minimum of 2 μg of total RNA (per sample) following the manufacturer’s instructions (Illumina Inc., San Diego, CA) and 150 base pair paired-ends were sequenced on an Illumina platform. Illumina RNA-seq reads of the nematode were then mapped against the Rv genome of *R*. *similis* [24] using the same parameters as described above. Gene expression patterns were deduced from the aligned reads and determined as FPKM values. Public RNA-seq libraries generated from different nematode stages (eggs, juveniles, females and males) were also used to estimate transcripts abundance (FPKM values) of all effector genes identified and presented as heatmaps (SRA BioProject PRJNA427497 [23]).

### Generation of PVX-based constructs and transient expression *in planta*

To express different cell wall-degrading coding genes *in planta*, EcoRV-flanked PCR products of transcripts encoding the full protein (with signal peptide) were cloned into the pCR II-TOPO vector (Thermo Fisher Scientific, Waltham, MA, USA) according to the manufacturer’s directions and verified by automated Sanger sequencing. The corresponding plasmids were digested with the EcoRV restriction enzyme, gel purified, and subcloned into the EcoRV-linearized PVX-based vector pP2C2S (obtained from D. Baulcombe, Sainsbury Laboratories, Norwich, England). The integrity of all PVX clones was verified by automated sequencing. The pP2C2S plasmids were linearized with SpeI and capped transcripts were generated from the cDNA clones using the Ambion T7 mMessage Machine kit (Thermo Fisher Scientific). Transcripts of each individual PVX construct were mechanically rub-inoculated into fully expanded leaves of *Nicotiana benthamiana* plants. Transcripts were also produced from pP2C2S plasmids without inserts (“empty” PVX vector) and rub-inoculated into *N*. *benthamiana* plants to serve as controls representing a wild-type PVX infection (PVX/WT). Plants inoculated with buffer were used as negative (“healthy”) controls. Inoculated plants were monitored daily for symptoms. For each inoculation experiments a minimum of three plants were inoculated per construct and repeated three times. At 14 DAI, the leaves were photographed, collected, snap frozen in liquid nitrogen, and stored at ^_^80°C until RNA extraction.

### Phylogenetic analyses

To infer the phylogeny of the different gene/gene families (i.e. “bacterial extracellular solute-binding protein” and GH62), homologs of nematode genes and closest organisms were searched in public databases (nr database at NCBI or from Wormbase) and the top 100 BLAST hits (e-value < e^-5^ and bit score >50) were collected. For the phylogenetic reconstruction of different plant-parasitic nematodes within the phylum Nematoda, eighty-six CEGMA genes conserved in the genome and/or transcriptome resources of 15 nematodes species described above were used. Multiple sequence alignments were done with MUSCLE [86]. Alignments were then concatenated and model selection for each partition was carried out using the IQtree server. A concatenated multi-gene phylogeny was generated using the ultra-fast mode and 1,000 bootstrap replicates [87].

## Supporting information

S1 Table List of 2,218 *Radopholus similis* transcripts coding predictive secreted proteins and corresponding annotation. (XLSX)

S2 Table List of 540 *Radopholus similis* transcripts coding predictive secreted proteins with representation in the gland cells library. Transcript abundance was determined as Reads Per Kilobase of Transcript per Million mapped reads (FPKM) values. Presence/absence of homologues in other nematode species were performed by BLAST analyses (e-value < 10^−5^ and bit score > 50).
(XLSX)

S3 Table List of candidate effector genes of *Radopholus similis* used for promoter motif discover. This list comprises genes herein validated in the nematode esophageal glands, as well as homologues of known effector genes of other plant-parasitic nematodes.
(XLSX)

S4 Table List of 109 genes coding predictive secreted proteins associated with the subventral gland cell Rs-SUG box in *Radopholus similis*. (XLSX)

S5 Table List of primers used in this study. (XLSX)

S1 Fig Maximum-likelihood phylogenetic tree of *Radopholus similis* predicted “bacterial extracellular solute-binding protein” (PF13343) with representative proteins of other organisms. Phylogenetic groups are colored in agreement to their taxonomy. Nematode representative sequences were obtained from the root lesion nematode *Pratylenchus penetrans*, the cyst nematodes *Heterodera avenae*, *H*. *glycines*, and *Globodera rostochiensis*. All bacteria, fungi and Arthropoda sequences were retrieved from the nr database at NCBI. Bootstraps values are shown at the nodes.
(TIF)

S2 Fig Multiple sequence alignment of the predicted Rs-GH62 protein of *Radopholus similis* with close GH62 protein sequences from bacteria. Species of bacteria were chosen based on the phylogenetic tree presented in Fig 6B. Sequence logo correspond to the consensus sequence of the corresponding alignment. The predicted signal peptide and corresponding GH62 and CBM_2 domains are presented in different colors as shown in the legend.
(TIF)

S3 Fig Alignment of Rs-GH62 genomic DNA and cDNA sequences of *Radopholus similis*. Exons and introns are indicated by green and red arrows, respectively. The genomic DNA and cDNA sequences were cloned using nematodes from the Hawaii population (indicated by an asterisk). All the remaining sequences were obtained from the draft genome assemblies of different geographical populations of *R*. *similis*.
(TIF)

S4 Fig Phenotypic changes in *Nicotiana benthamiana* plants infected with recombinant PVX/Rs-PEL recombinant virus. **(A-B)** Plants expression the recombinant PVX/Rs-PEL virus exhibited a strong necrotic reaction and general decline of health **(A)**, including a reduced and necrotic root system **(B)** at 21 days after infection.
(TIF)

S5 Fig RT-PCR detection of *Radopholus similis* candidate effectors in systemically infected leaves of *Nicotiana benthamiana* plants. Leaves of three different plants infected with recombinant PVX vectors were collected for RNA extraction at 14 days after inoculation: **(A)** PVX/Rs-PEL; **B)** PVX/Rs-GH16; **C)** PVX/Rs-EXPB; **D)** PVX/Rs-GH62. As control three different plants were inoculated with wild-type PVX transcripts (PVX/WT). For RT-PCR validation three sets of primers were used: 1) primers specific for each corresponding *R*. *similis* gene; 2) primers for the PVX coat protein for confirmation of PVX infection; and 3) primers for the *tubulin 1* gene of *N*. *benthamiana* were used to validate plant cDNA synthesis. DNA marker: 1 Plus kb DNA ladder (Thermo Fisher Scientific). P1-P3: leaves collected from three independently-inoculated plants.
(TIF)
