# Supplementary figures and images for: Targeted transcriptomics reveals signatures of large-scale independent origins and concerted regulation of effector genes in Radopholus similis
Source: PLoS Pathog. 2021 Nov 8;17(11):e1010036. doi: 10.1371/journal.ppat.1010036 (PMC8601627; doi:10.1371/journal.ppat.1010036)

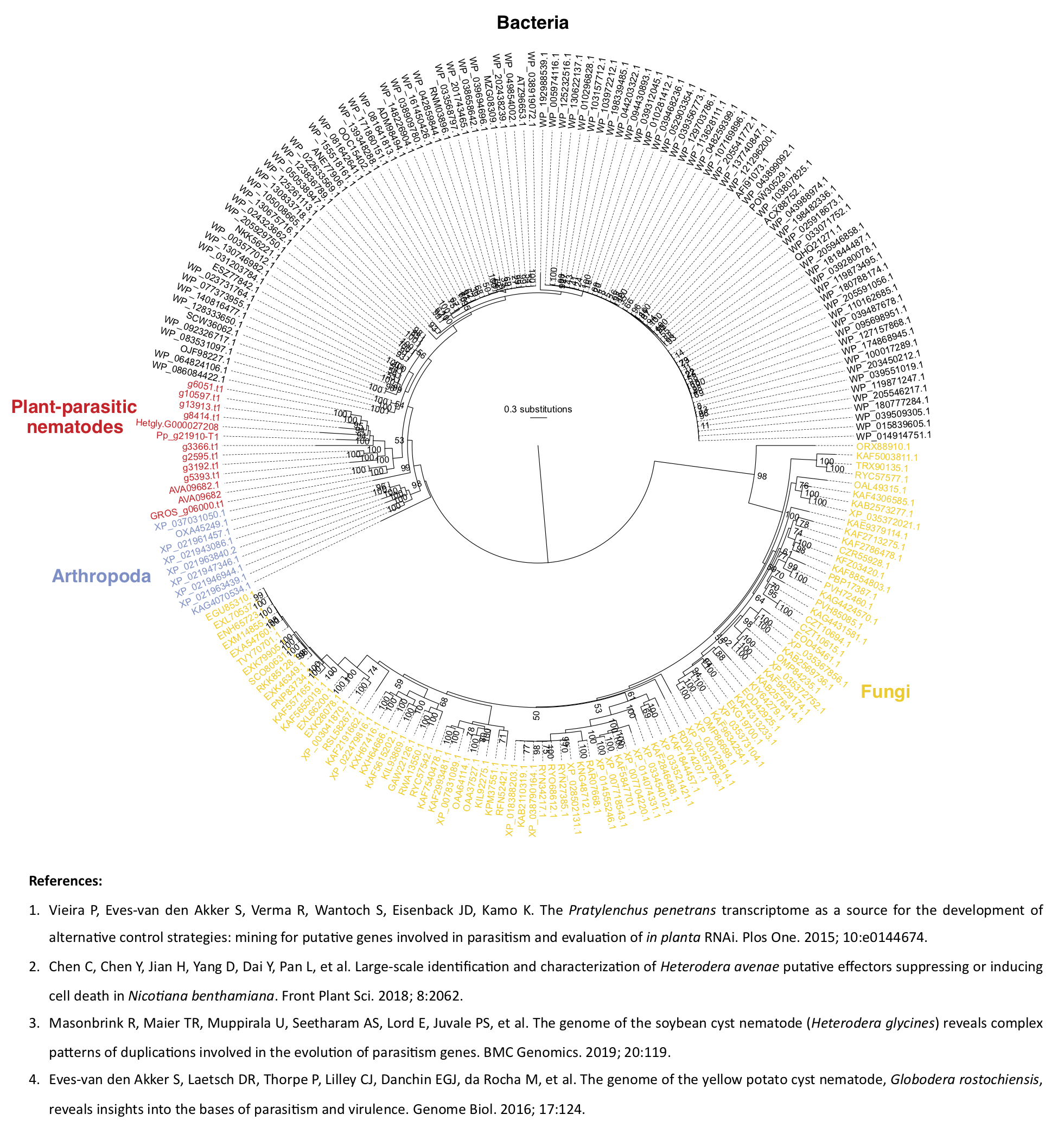

Supplement: S1 Fig — Phylogenetic groups are colored in agreement to their taxonomy. Nematode representative sequences were obtained from the root lesion nematode Pratylenchus penetrans, the cyst nematodes Heterodera avenae, H. glycines, and Globodera rostochiensis. All bacteria, fungi and Arthropoda sequences were retrieved from the nr database at NCBI. Bootstraps values are shown at the nodes. (TIF) [file ppat.1010036.s006.tif]

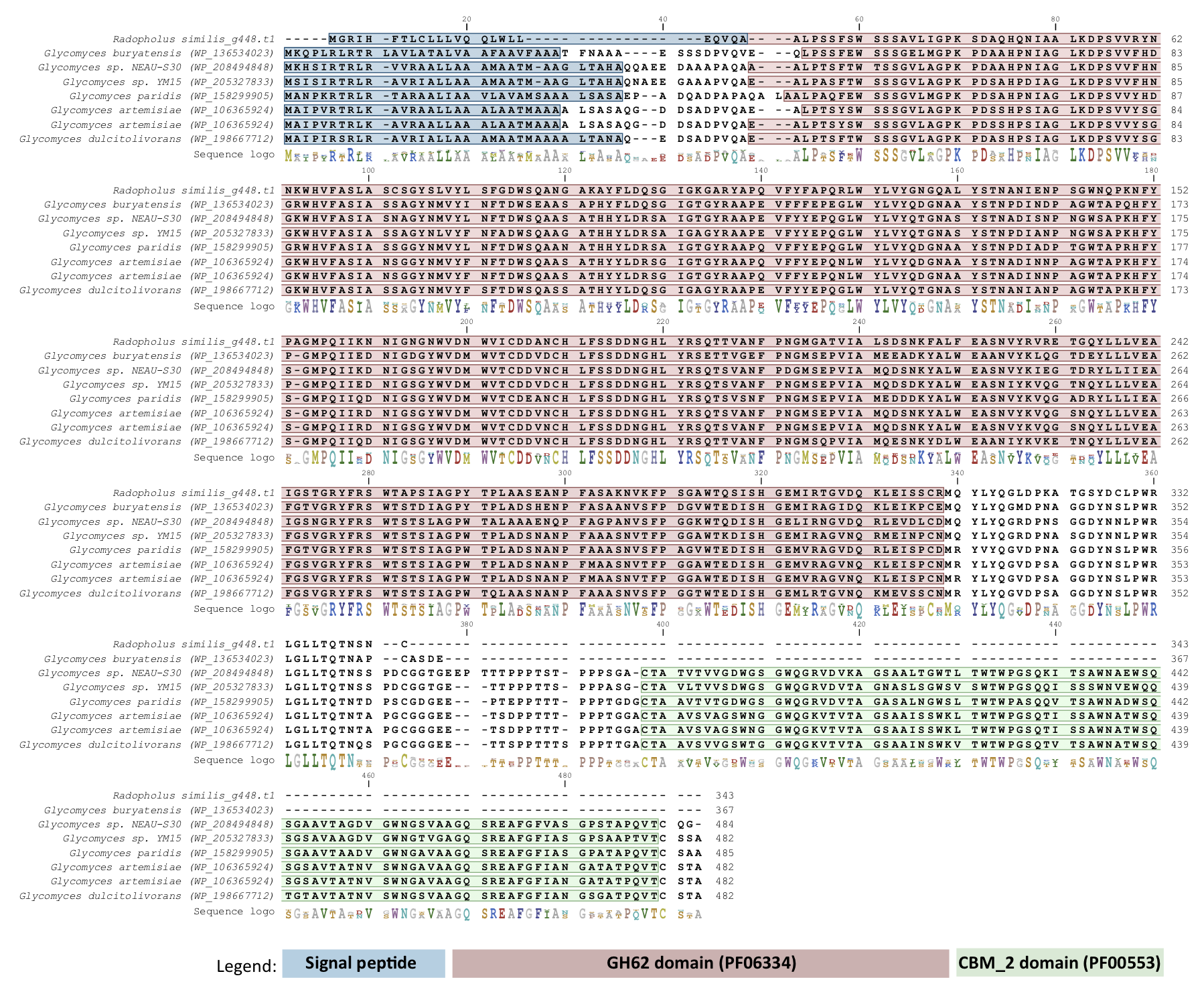

Supplement: S2 Fig — Species of bacteria were chosen based on the phylogenetic tree presented in Fig 6B. Sequence logo correspond to the consensus sequence of the corresponding alignment. The predicted signal peptide and corresponding GH62 and CBM_2 domains are presented in different colors as shown in the legend. (TIF) [file ppat.1010036.s007.tif]

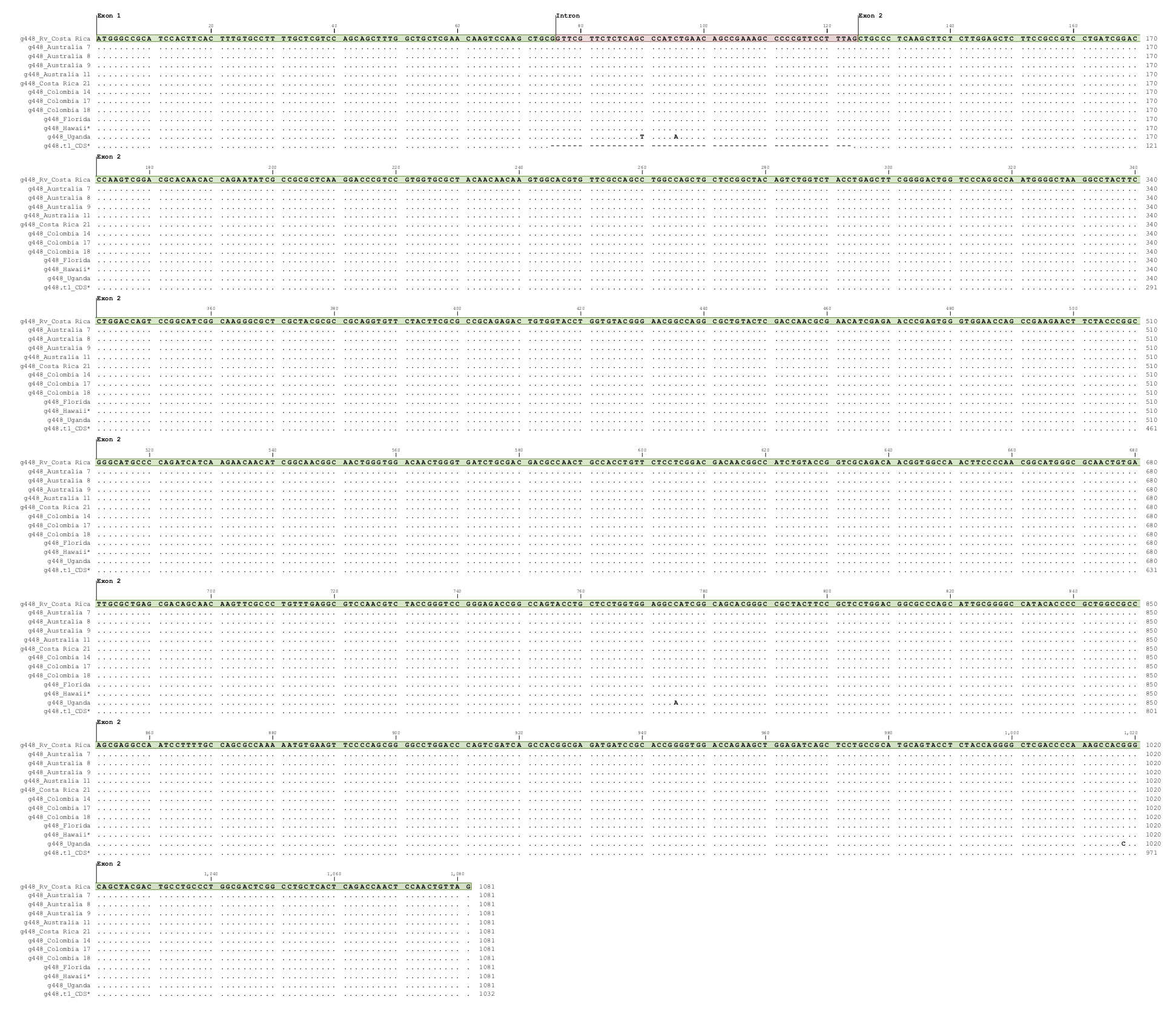

Supplement: S3 Fig — Exons and introns are indicated by green and red arrows, respectively. The genomic DNA and cDNA sequences were cloned using nematodes from the Hawaii population (indicated by an asterisk). All the remaining sequences were obtained from the draft genome assemblies of different geographical populations of R. similis. (TIF) [file ppat.1010036.s008.tif]

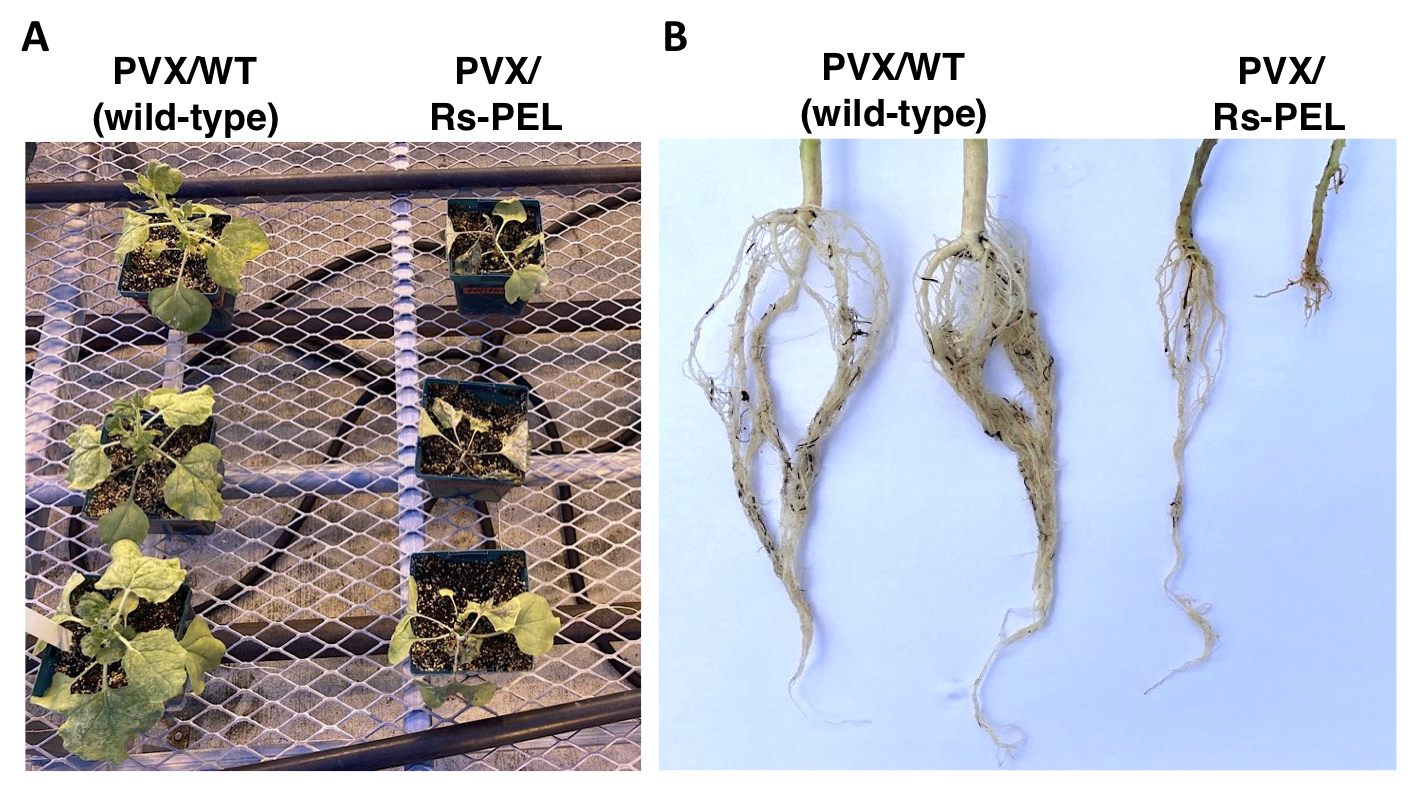

Supplement: S4 Fig — (A-B) Plants expression the recombinant PVX/Rs-PEL virus exhibited a strong necrotic reaction and general decline of health (A), including a reduced and necrotic root system (B) at 21 days after infection. (TIF) [file ppat.1010036.s009.tif]

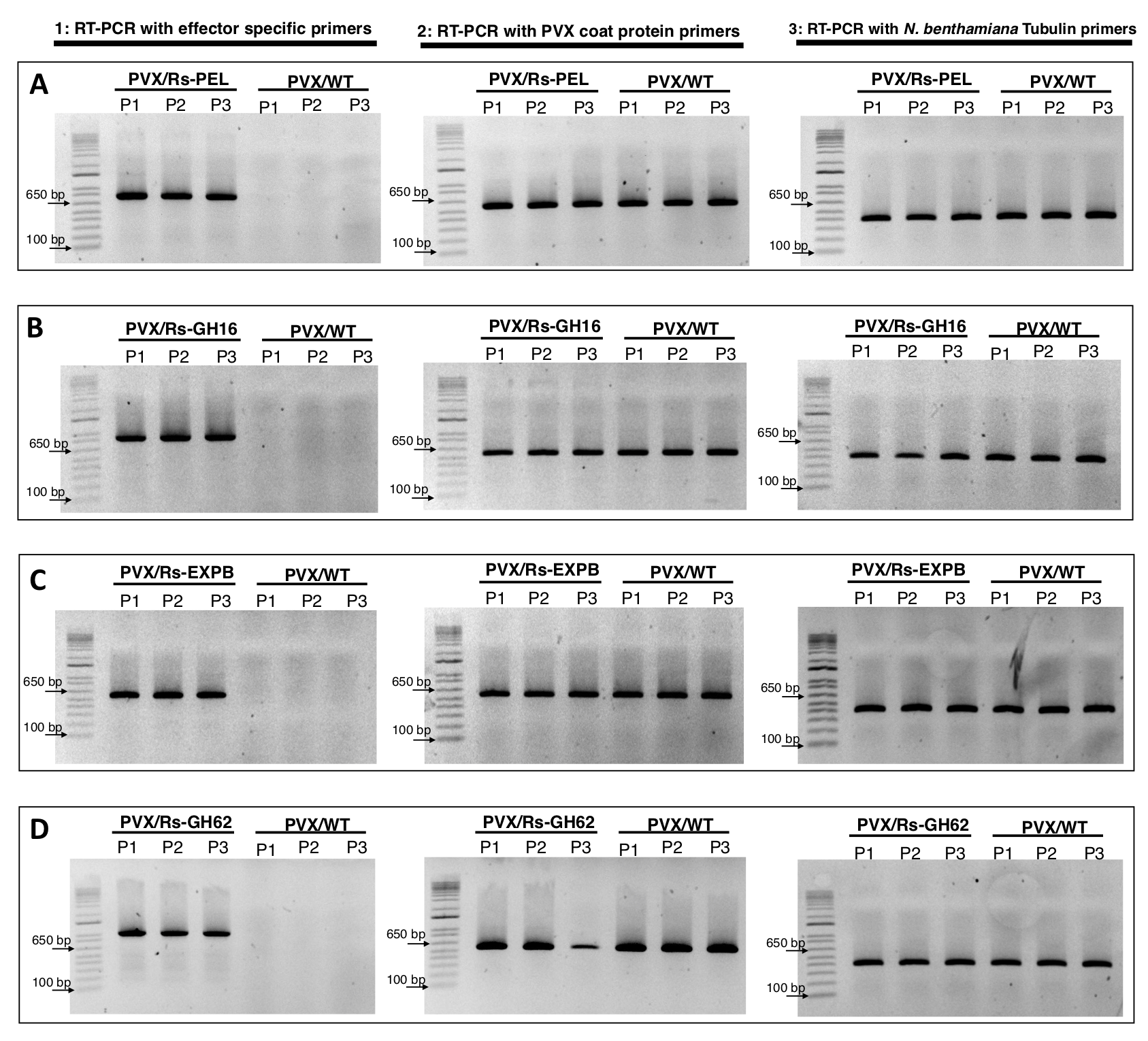

Supplement: S5 Fig — Leaves of three different plants infected with recombinant PVX vectors were collected for RNA extraction at 14 days after inoculation: (A) PVX/Rs-PEL; B) PVX/Rs-GH16; C) PVX/Rs-EXPB; D) PVX/Rs-GH62. As control three different plants were inoculated with wild-type PVX transcripts (PVX/WT). For RT-PCR validation three sets of primers were used: 1) primers specific for each corresponding R. similis gene; 2) primers for the PVX coat protein for confirmation of PVX infection; and 3) primers for the tubulin 1 gene of N. benthamiana were used to validate plant cDNA synthesis. DNA marker: 1 Plus kb DNA ladder (Thermo Fisher Scientific). P1-P3: leaves collected from three independently-inoculated plants. (TIF) [file ppat.1010036.s010.tif]
